# m^6^A epitranscriptome analysis reveals differentially methylated transcripts that drive early chemoresistance in bladder cancer

**DOI:** 10.1093/narcan/zcad054

**Published:** 2023-11-16

**Authors:** Emmanuelle Hodara, Aubree Mades, Lisa Swartz, Maheen Iqbal, Tong Xu, Daniel Bsteh, Peggy J Farnham, Suhn K Rhie, Amir Goldkorn

**Affiliations:** Division of Medical Oncology, Department of Medicine, Keck School of Medicine of USC and Norris Comprehensive Cancer Center, Los Angeles, CA 90033, USA; Division of Medical Oncology, Department of Medicine, Keck School of Medicine of USC and Norris Comprehensive Cancer Center, Los Angeles, CA 90033, USA; Division of Medical Oncology, Department of Medicine, Keck School of Medicine of USC and Norris Comprehensive Cancer Center, Los Angeles, CA 90033, USA; Division of Medical Oncology, Department of Medicine, Keck School of Medicine of USC and Norris Comprehensive Cancer Center, Los Angeles, CA 90033, USA; Division of Medical Oncology, Department of Medicine, Keck School of Medicine of USC and Norris Comprehensive Cancer Center, Los Angeles, CA 90033, USA; Division of Medical Oncology, Department of Medicine, Keck School of Medicine of USC and Norris Comprehensive Cancer Center, Los Angeles, CA 90033, USA; Department of Biochemistry and Molecular Medicine, Keck School of Medicine of USC, Los Angeles, CA 90033, USA; Department of Biochemistry and Molecular Medicine, Keck School of Medicine of USC, Los Angeles, CA 90033, USA; Division of Medical Oncology, Department of Medicine, Keck School of Medicine of USC and Norris Comprehensive Cancer Center, Los Angeles, CA 90033, USA; Department of Biochemistry and Molecular Medicine, Keck School of Medicine of USC, Los Angeles, CA 90033, USA

## Abstract

*N* ^6^-Methyladenosine (m^6^A) RNA modifications dynamically regulate messenger RNA processing, differentiation and cell fate. Given these functions, we hypothesized that m^6^A modifications play a role in the transition to chemoresistance. To test this, we took an agnostic discovery approach anchored directly to chemoresistance rather than to any particular m^6^A effector protein. Specifically, we used methyl-RNA immunoprecipitation followed by sequencing (MeRIP-seq) in parallel with RNA sequencing to identify gene transcripts that were both differentially methylated and differentially expressed between cisplatin-sensitive and cisplatin-resistant bladder cancer (BC) cells. We filtered and prioritized these genes using clinical and functional database tools, and then validated several of the top candidates via targeted quantitative polymerase chain reaction (qPCR) and MeRIP-PCR. In cisplatin-resistant cells, SLC7A11 transcripts had decreased methylation associated with decreased m^6^A reader YTHDF3 binding, prolonged RNA stability, and increased RNA and protein levels, leading to reduced ferroptosis and increased survival. Consistent with this, cisplatin-sensitive BC cell lines and patient-derived organoids exposed to cisplatin for as little as 48 h exhibited similar mechanisms of SLC7A11 upregulation and chemoresistance, trends that were also reflected in public cancer survival databases. Collectively, these findings highlight epitranscriptomic plasticity as a mechanism of rapid chemoresistance and a potential therapeutic target.

## Graphical Abstract

**Graphical Abstract:**
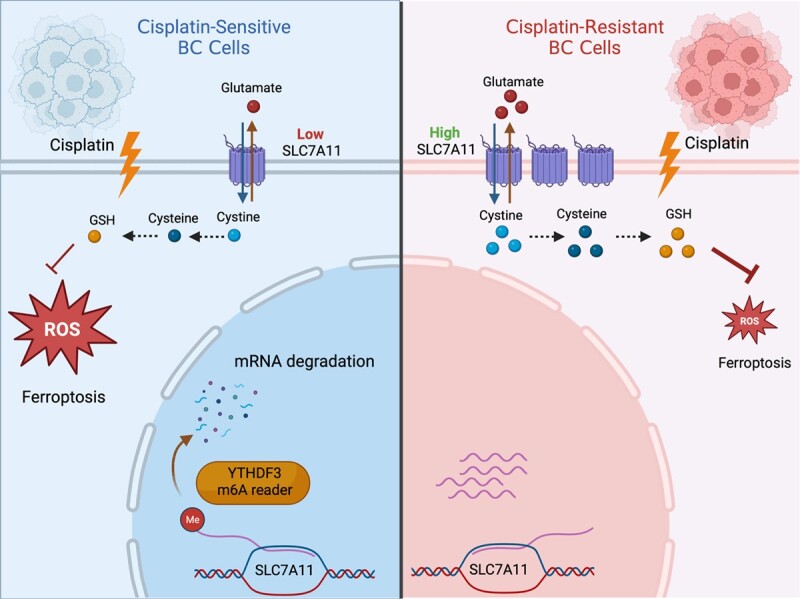

## Introduction

Cancer treatment has advanced dramatically in recent decades, but the majority of patients with advanced malignancies ultimately succumb to their disease. Urinary bladder cancer (BC) takes a major societal toll in the United States, with an estimated 82 290 annual new cases in 2022. The backbone of therapy for locally advanced and metastatic BC is cisplatin chemotherapy, but a majority of tumors develop resistance, resulting in 16 710 deaths in 2022 (1). Treatment resistance traditionally has been attributed to rare genetically resistant subpopulations of cells in the original tumor or to adaptive genomic alterations induced by drug treatment propagated via Darwinian selection (2). However, we and others have demonstrated that within a genetically identical cell population, significant epigenetic and transcriptional heterogeneity can exist or arise by phenotypic plasticity in response to environmental factors and contribute to drug resistance (3–7).

Another mechanism of phenotypic plasticity involves *N*^6^-methyladenosine (m^6^A) RNA modification, which has been shown to dynamically regulate messenger RNA (mRNA) processing, differentiation and cell fate (8–12). m^6^A is deposited co-transcriptionally by a conserved core writer complex of METTL3, METTL14 and WTAP (13,14), whereas m^6^A is removed by two demethylases (erasers), ALKBH5 and FTO (15,16), although the precise functions ascribed to these proteins continue to evolve. m^6^A modifications are recognized by binding proteins (readers), namely YTHDF1–3, which serve as effectors of downstream sequelae. m^6^A has a major effect on splicing in a small number of transcripts (17), influences epigenetic silencing and—most importantly—affects mRNA stability (18–21). Although m^6^A status is not universally malleable (22), it can vary by cell type, explaining tissue-specific patterns of expression (23). Moreover, m^6^A-containing mRNAs are believed to be key regulators of specific signaling pathways (22). For example, m^6^A residues are found on mRNAs encoding tumor suppressor *APC*, and m^6^A-mediated degradation of *APC* leads to cancer progression (24). Indeed, deregulation of m^6^A and its effector proteins (writers, erasers, readers) has been implicated in cancer initiation, progression, drug resistance and relapse in a variety of malignancies (10).

In BC, one analysis of The Cancer Genome Atlas (TCGA) database showed that 80% of BC samples were altered in one or more m^6^A effectors, most commonly VIRMA, YTHDF1/3, METTL4 and RBM15 (25). Another study demonstrated that the m^6^A eraser, ALKBH5, inhibited cell proliferation and sensitized BC cells to cisplatin (26). More recently, a third study reported that circular RNA *circ0009399* binds WTAP to modulate expression of target RNA through m^6^A and reduce cisplatin sensitivity in BC (27). These studies underscore the potential impact of elucidating m^6^A’s role in BC, calling for a more systematic analysis to identify epitranscriptomic drivers of disease progression and drug resistance.

To address this need, in this study we set out to systematically identify changes in m^6^A RNA methylation and RNA expression between cisplatin-sensitive and cisplatin-resistant BC cells. Using unbiased transcriptome-wide m^6^A profiling followed by targeted validation, we demonstrated that m^6^A modifications regulate expression of clinically relevant gene transcripts in BC, and that a subset of these modifications may promote resistance to chemotherapy. Specifically, m^6^A modification regulated the levels of SLC7A11, a cystine–glutamate antiporter that potentiates cisplatin resistance in BC. Decreased m^6^A levels on SLC7A11 mRNA led to decreased binding of the m^6^A reader YTHDF3, decreased mRNA degradation and increased SLC7A11 expression in cisplatin-resistant cells compared to cisplatin-sensitive cells. The same trend was observed with short-term cisplatin treatment, suggesting that m^6^A RNA modifications can regulate early phenotypic changes that promote transition to cisplatin resistance in BC.

## Materials and methods

### Cell culture

Human BC cell lines, T24 and UM-UC-3, were cultured in RPMI 1640 and Dulbecco’s modified Eagle medium (DMEM; Mediatech Inc., Manassas, VA), respectively, supplemented with 10% heat-inactivated fetal bovine serum (FBS; Omega) and 1% penicillin/streptomycin (100 units/ml, Invitrogen), at 37°C and 5% CO_2_. Prior to conducting all experiments, the cell lines were authenticated using nine-marker short tandem repeat profiling and testing interspecies and mycoplasma contamination (CellCheck 9 Plus, IDEXX BioAnalytics, Columbia, MO). We maintain stringent, good cell culture practice and keep extensive records of cell lines’ culture and harvesting conditions, including passage count and cell density. We performed all experiments below passage 20, after which cultures were restarted using thawed cells from earlier passages. T24R2 cell lines were generated by serial desensitization of T24 cells with cisplatin and were a generous gift from the Byun Lab (Seoul National University Bundang Hospital) (28). T24R2 cell lines were maintained in RPMI 1640 media with 6.6 μM cisplatin (Cat# NSC 119875, Selleck Chemicals, Houston, TX).

### Organoid culture

CK-9151 is a patient-derived BC organoid acquired from the National Cancer Institute (NCI) Patient-Derived Models Repository (PDMR; model 772611-094-R-V3-organoid, sample number CK9151). CTC-1044 is a patient-derived BC organoid generated in our laboratory. Through an Institutional Review Board-approved protocol and under informed consent, we received excess tissue not needed for diagnostic purposes from transurethral resection of bladder tumor (TURBT) from patients with transitional cell carcinoma of the bladder undergoing treatment at the Keck School of Medicine of USC. Cells were treated with Dispase II (1.5 mg/ml, Cat# 17105-041, Thermo Fisher) in DMEM/F12 (Cat# 12634028, Thermo Fisher) for 2 h at 37°C to break down the basement membrane. Cells were then collected, pelleted by centrifugation at 300 × *g* for 5 min and resuspended in 35 µl BME (Cat# 3533-001-02, Cultrex) per well and plated in a pre-warmed 24-well plate. The plate was inverted and incubated at 37°C for 15 min. Once the BME solidified, human bladder organoid medium (BOM) was added based on a modified 6A Media Recipe provided by the NCI PDMR with the following concentrations: advanced DMEM/F12 (1×) (Invitrogen, Cat# 12634-028), HEPES (10 mM) (Invitrogen, Cat# 15630080), GlutaMax Supplement (1×) (Life Technologies, Cat# 35050061), Primocin (0.1 mg/ml) (InvivoGen, Cat# Ant-pm-2), L-WRN Conditioned Media (50%) (Sigma–Aldrich, Cat# SCM105), *N*-acetylcysteine (1.25 mM) (Sigma, Cat# A9165-5G), nicotinamide (10 mM) (Sigma, Cat# N0636-100G), B-27 Supplement (1×) (Life Technologies, Cat# 17504044), N-2 Supplement (1×) (Life Technologies, Cat# 17502048), Y-27632 dihydrochloride (10 μM) (Tocris, Cat# 1254), FGF10 (100 ng/ml) (Peprotech, Cat# 100-26), FGF7 (25 ng/ml) (Peprotech, Cat# 100-19), FGF2 (12.5 ng/ml) (Peprotech, Cat# 100-18B) and A83-01 (5 μM) (Tocris, Cat# 2939). The medium was filtered using a 0.22 μm filter and prepared fresh every 3 weeks. Organoids were used for experiments 7–10 days after passaging and were passaged every 21 days.

### RNA isolation

Total RNA was extracted from cell lines using TRIzol reagent and Direct-zol RNA extraction kit (R2071, Zymo Research), which included treatment with DNase I for 20 min at 37°C. Total RNA was extracted from organoids using the RNeasy Micro Kit (Cat# 74004, Qiagen, Germany). The concentration of total RNA was measured by Qubit RNA HS Assay Kit (Cat# Q32855, Thermo Fisher Scientific) or via nanodrop, depending on the starting amount.

### RNA fragmentation

Total RNA was chemically fragmented into ∼200 nt fragments as previously described (29). Briefly, 3–5 μg of purified RNA was adjusted to a volume of 18 μl with RNase-free water. Two microliters of 10× RNA Fragmentation Buffer (100 mM Tris–HCl, 100 mM ZnCl_2_ in nuclease-free H_2_O) was added and incubated in a preheated thermal cycler for 4–5 min at 70°C. The reaction was stopped by adding 2 μl of 0.5 M ethylenediaminetetraacetic acid. The following were then added to the mixture: 178 μl of H_2_O, 20 μl of sodium acetate (3 M, pH 5.2, S7899, Sigma–Aldrich, St Louis, MO), 14.4 μl of glycogen (5 mg/ml, Cat# AM9510, Thermo Fisher Scientific) and 500 μl of 100% ethanol and incubated at −80°C overnight. Fragmented RNA was pelleted by centrifuge (30 min at 12 000 × *g* at 4°C), washed once with 75% ethanol and resuspended in RNase-free water (10 μl H_2_O per 1 μg human total RNA). The size distribution was assessed using RNA 6000 Pico Kit on BioAnalyzer (Cat# 50671513, Agilent Technologies, Santa Clara, CA).

### MeRIP

Methyl-RNA immunoprecipitation (MeRIP) was performed as previously published (29). Briefly, 30 μl of Protein A magnetic beads (Cat# 10002D, Thermo Fisher Scientific) and 30 μl of Protein G magnetic beads (Cat# 10004D, Thermo Fisher Scientific) were washed twice with 500 μl IP buffer (150 mM NaCl, 10 mM Tris–HCl, pH 7.5, 0.1% IGEPAL CA-630 in nuclease-free H_2_O) and resuspended in 500 μl IP buffer, and tumbled with 5 μg anti-m^6^A antibody (Cat# E1610, NEB, Ipswich, MA) at 4°C overnight. The bead–antibody mixture was washed two more times with 500 μl IP buffer and resuspended in 500 μl IP buffer containing the fragmented RNA, 100 μl of 5× IP buffer and 5 μl RNasin Plus RNase Inhibitor (Cat# N2611, Promega, Madison, WI), and incubated for 2 h at 4°C.

The RNA reaction mixture was washed twice with 1000 μl IP buffer, twice with 1000 μl low-salt IP buffer (50 mM NaCl, 10 mM Tris–HCl, pH 7.5, 0.1% IGEPAL CA-630 in nuclease-free H_2_O) and twice with 1000 μl high-salt IP buffer (500 mM NaCl, 10 mM Tris–HCl, pH 7.5, 0.1% IGEPAL CA-630 in nuclease-free H_2_O) for 10 min each at 4°C. After the washes, the m^6^A-enriched fragmented RNA was eluted from the beads in 200 μl of RLT buffer supplied in the RNeasy Micro Kit (Cat# 74004, Qiagen, Germany) for 2 min at room temperature. Magnetic separation rack (Cat# 1231D, Thermo Fisher Scientific) was applied to pull beads to the side of the tube. Supernatant was collected to a new tube and combined with 400 μl of 100% ethanol. The mixture was transferred to an RNeasy MicroElute spin column (RNeasy Micro Kit) and centrifuged at >12 000 rpm at 4°C for 1 min. The column membrane was washed with 500 μl RPE buffer (RNeasy Micro Kit) once and with 500 μl of 80% ethanol once. The column was centrifuged at full speed for 5 min at 4°C to remove residual ethanol. The m^6^A-enriched RNA was eluted with 14 μl nuclease-free water.

### RT-qPCR and MeRIP-qPCR

Complementary DNA (cDNA) synthesis was performed using qScript cDNA SuperMix (Cat# 95408-500, QuantaBio, Beverly, MA). Real-time polymerase chain reaction (PCR) was performed using PerfeCTa SYBR Green FastMix (Cat# 95071-250, QuantaBio) using a Bio-Rad CFX96 Real-Time PCR Detection System. For MeRIP samples, 2 µl of 14 µl eluted RNA was reverse transcribed. MeRIP efficiency was assessed by SETD7/GAPDH reverse transcription-quantitative PCR (RT-qPCR) using a threshold of 100-fold enrichment. The expression percentage of a target gene in RNA immunoprecipitation (RIP) sample relative to the input control sample was determined as follows: %input = 2^(Ct of target gene in input control − Ct of target gene in RIP). The relative gene expression of various target genes after altering global m^6^A (using genetic and pharmacological modulation) was calculated using GAPDH as a housekeeping gene. Relative expression = 2^(Ct of GAPDH − Ct of target gene). Primer sequences can be found in Supplementary Table S1. All experiments were performed in biological triplicates, and additional technical triplicates were used for all RT-qPCR experiments.

### Library preparation and sequencing

RNA sequencing (RNA-seq) libraries were prepared using NEBNExt Ultra II RNA Library Prep Kit for Illumina (Cat# E7420, NEB) according to the manufacturer’s protocol. MeRIP libraries were prepared using SMARTer Stranded Total RNA-Seq Kit v2—Pico Input Mammalian (Cat# 634413, Takara-Clontech, Japan) according to manufacturer’s instructions. Briefly, 3.5 µl of 14 µl eluted RNA and 50 ng input RNA were used for library construction omitting the fragmentation step. Libraries for IP RNA and input RNA were PCR amplified for 16 and 12 cycles, respectively. Purified libraries were quantified using Qubit dsDNA High Sensitivity Kit (Cat# Q32851, Thermo Fisher) using a Qubit fluorometer, and the size distribution was checked by BioAnalyzer (Agilent Technology) using Agilent High Sensitivity DNA Kit (Cat# 5067-4626, Agilent). The samples were then sequenced using a NovaSeq PE 150 (Illumina, San Diego, CA). Adapter sequences were removed, and sequences were demultiplexed using the bcl2fastq software (Illumina).

### RNA-seq analysis

RNA-seq reads were aligned to human genome hg38 with reference annotation GENCODE v39 and counted using STAR (version 2.7.0) (30,31). Only uniquely mapped reads without duplicates were selected using samtools (version 1.10) (32). Read counts were assigned to genes using Subread featureCounts (33). Read counts were normalized using DESeq2 package in R (version 4.1.3). To generate more accurate log_2_ fold change (LFC) estimates, shrinkage of the LFC estimates toward zero was applied using DESeq2 (34). Differentially expressed transcripts with absolute |log_2_FC| > 0.5 and adjusted *P*-value <0.05 were retained. Gene set enrichment analysis (GSEA) and Gene Ontology (GO) analysis were implemented and visualized using clusterProfiler package (35–37).

### Differential m^6^A analysis

MeRIP-seq (MeRIP followed by sequencing) reads were aligned to human genome hg38 with reference annotation GENCODE v39 and counted using STAR (version 2.7.0) (30,31). Only uniquely mapped reads without duplicates were selected using samtools (version 1.10) (32). IP over input peaks were called using MACS2 callpeak using the parameters ‘-nomodel -extsize100 -gsize300e6’ (38). Differential m^6^A analysis was performed using DEQ package in R as previously published (39). Briefly, DEQ runs statistical analysis using DESeq2, edgeR and QNB packages (34,39–41). Results that were statistically significant with adjusted *P*-value <0.05 using all three packages were considered significant. Gene and peak expression changes were estimated as LFCs from DESeq2. Additional filtering to mitigate the confounding effect of differential gene expression on determining differential methylation was applied using |peak IP log_2_FC − gene input log_2_FC| ≥ 1. Peaks with <10 read counts were also removed to mitigate the confounding effect of differential gene expression (see the ‘Results’ section for further discussion). Peaks were annotated with gene name and gene location using DEQ.

### siRNA knockdown

Dicer-substrate short interfering RNAs (DsiRNAs) targeting transcripts of interest and corresponding negative control were purchased from Integrated DNA Technologies (Coralville, IA). DsiRNA sequences, concentrations and time points used can be found in Supplementary Table S2.

*Cancer cell lines*: T24, T24R2 and UM-UC-3 cells; transfection was performed 24 h after seeding. Lipofectamine 3000 (Cat# L3000015, Invitrogen, Waltham, MA) and OPTI-MEM I (Cat# 31985062, Thermo Fisher) reagents were used to transfect cells at 70% confluence in either 96-well or 6-well plates according to the manufacturer’s protocol. Cells were harvested and total RNA was extracted at 24 or 48 h depending on the transcript. Knockdown efficiency was evaluated by RT-qPCR and western blot. For experiments conducted in 96-well plates, RNA was extracted using Power SYBR Green Cells-to-Ct Kit (Cat# 4402954, Invitrogen).

*Patient-derived organoids (PDOs)*: Transfections were performed in 24-well plates 10 days after passaging the organoids using Lipofectamine RNAiMAX (Cat# 13778030, Invitrogen) and OPTI-MEM I reagents. For each well, 45 µl RNAiMax and 30 µl OPTI-MEM I were incubated for 5 min before adding the corresponding siRNA. The siRNA complexes were then incubated for 20 min before making up the volume to 450 µl using BOM without antibiotics and supplemented with 10% FBS. The organoid medium was then replaced with the mixture. Organoids were harvested and total RNA was extracted 72 h after transfection.

### Cisplatin resistance assay

*Cancer cell lines*: T24, T24R2 or UM-UC-3 cells were seeded in 96-well plates and transfected the following day with corresponding siRNA or negative control for 24–48 h prior to treating the cells with 10 µM cisplatin (Cat# S1166, Selleck Chemicals) for 48 h. Cisplatin was dissolved in 154 mM NaCl saline and stored in single-use aliquots at −20°C. After 48 h, MTS proliferation assay was performed using CellTiter 96 AQueous One Solution Cell Proliferation Assay (Cat# G3582, Promega) according to the manufacturer’s protocol. Briefly, 20 µl of MTS solution was added to each well and incubated at 37°C for 2 h, after which absorbance at 490 nm was recorded using a 96-well plate reader. Cell viability was calculated as follows: percentage viability = (absorbance[sample]/absorbance[NC − cisplatin]) × 100%. Coefficient of drug interaction (CDI) to evaluate synergy was calculated as follows: CDI = percentage viability[condition + cisplatin]/(percentage viability[condition − cisplatin] × percentage viability[NC − cisplatin]). CDI < 1.0 indicates synergy and CDI < 0.7 indicates significant synergy (42). For each sample, six technical replicates were used to calculate averages. Each experiment was repeated in three to four biological replicates.

*PDOs*: CK9151 or CTC-1055 were passaged into 24-well plates and treated 7–10 days with 50 µM cisplatin for 48 h alone or transfected with siSLC7A11for 48 h followed by treatment with 50 µM cisplatin for 48 h. After the treatment, organoids were digested with Dispase II (1.5 mg/ml) for 2 h at 37°C, followed by 15 min incubation with TrypLE (Cat# 12-605-010, Gibco) to yield single-cell resuspension. Cells were then counted using Trypan Blue Exclusion Assay using Nikon Eclipse TS100 Inverted Routine Microscope and Life Technologies Countess II FL Automated Cell Counter. Cell counts were used to calculate cell survival and CDI to evaluate synergy. Three technical replicates were performed for each sample, and the experiments were repeated in three biological replicates.

### Actinomycin D treatment

T24, T24R2 and UM-UC-3 cells were seeded in six-well plates with and without 10 µM cisplatin. After 48 or 72 h of treatment, cells were washed and collected at *t* = 0 in RLT buffer supplied in the RNeasy Micro Kit. The remainder of the wells were treated with 30 µl of actinomycin D (Cat# 11805017, Gibco) at a concentration of 1 mg/ml for a working concentration of 10 µg/ml in 3 ml of culture media. Actinomycin D was dissolved to 1 mg/ml in dimethyl sulfoxide and stored in single-use aliquots at −20°C. Samples were collected at *t* = 2, 4 and 6 h. RNA was extracted using the RNeasy Micro Kit, followed by cDNA synthesis and RT-qPCR analysis of transcripts of interest to calculate half-life of each transcript. Statistical significance was assessed by two-way analysis of variance (ANOVA).

### Lipid peroxidation detection

Lipid peroxidation was detected using C11-BODIPY (581/591) dye (Cat# D3861, Fisher Scientific). T24, T24R2 and UM-UC-3 cells were incubated with 5 mM C11-BODIPY in 24-well plates with and without 10 µM cisplatin treatment for 48 or 72 h. All cells were collected, spun down, washed and resuspended in Hanks’ balanced salt solution with calcium and magnesium (Cat# 14025092, Thermo Fisher), and analyzed using FACS on a BD SORP FACSYMPHONY S6 cell sorter. At least 10 000 events were recorded. All samples were performed in technical triplicates and experiments were repeated in three biological replicates.

### Western blotting

Western blots were performed as previously described (43). Briefly, whole cell lysates were extracted from human BC cells and BC PDOs using a RIPA lysis buffer (Cat# R0278, Sigma–Aldrich, St Louis, MO). Total protein concentration was determined by Lowry assay using the BSA Protein Assay Kit (Cat# 5000002, Bio-Rad, Hercules, CA). Ten to twenty micrograms of protein lysate samples were boiled in loading buffer for 10 min before running on 4–20% Tris–glycine gradient gels (Cat# EC6021BOX, Invitrogen) along with Blue Prestained Protein Marker (11–250 kDa) (Cat# 59329, Cell Signaling Technology, Danvers, MA). Western transfer was performed using the iBlot Dry Blotting System (Thermo Fisher) and PVDF iBlot Transfer Stack (Cat# IB401031, Invitrogen). Membranes were blocked in Odyssey blocking buffer (Cat# 927-40000, LI-COR, Lincoln, NE) and incubated with primary antibodies overnight at 4°C. After three washes in 1× TBST + 0.10% Tween 20 buffer, membranes were incubated in corresponding secondary antibodies for 45 min at room temperature. After three additional washes in 1× TBST + 0.10% Tween 20 buffer, membranes were visualized using an Odyssey DLx Imaging System (LI-COR). Digital images were processed and analyzed using ImageStudio version 5.0 software (LI-COR) and ImageJ was used to quantify protein expression. Primary and secondary antibodies and their corresponding concentrations can be found in Supplementary Table S3.

### Immunofluorescence

Cells were seeded in six-well plates containing microscopy coverslips. At collection time, cells were washed three times with 1× phosphate-buffered saline (PBS), fixed using 10% formalin for 15 min at 37°C and washed three times with 1× PBS. Cells were then permeabilized with 0.1% Triton X-100 in 1× PBS for 15 min at room temperature, washed three times with 1× PBS, blocked with 2% bovine serum albumin (BSA) in 1× PBS for 1 h at room temperature and incubated with primary antibody overnight at 4°C. After three washes with 1× PBS, cells were incubated with secondary antibody for 45 min at room temperature and washed three more times with 1× PBS. Coverslips were mounted on microscopy slides with VectaShield Antifade Mounting Medium with DAPI (Cat# H-1200-10, Vector Laboratories, Newark, CA) and sealed with nail polish.

Representative cells were selected and imaged on a Zeiss 800 Axio Imager.Z2 upright laser scanning confocal microscope, using an EC Plan-Neofluar 40×/1.30 oil lens. Images (1024 × 1024 pixels) were acquired using GaAsP-PMT detectors. Samples were illuminated with 405 and 488 nm lasers at 0.4% and 0.8%, respectively, with pinhole size set to 1 au. Alexa Fluor 488 acquisition parameters were as follows: 505–545 nm (detection wavelength range), 4.12 μs (pixel time) and 650 V (gain). For *z*-stack imaging, slices were acquired with 1 μm intervals. Image analysis was then performed uniformly using ImageJ. Primary and secondary antibodies and their corresponding concentrations can be found in Supplementary Table S4.

### Organoid histology staining

Organoids and tissue were fixed in 4% paraformaldehyde for 3 h, dehydrated and paraffin-embedded according to standard histology procedures. Sections were stained with hematoxylin and eosin and the following antibodies: Keratin 5 (AF138 COVANCE 160P-100), Ki67 (Monosan MONX10283), Keratin 20 (KS20.8 Dako M7019), TP63 (4A4 Abcam ab735) and Uroplakin III (AU1 Progen 651108) (44), according to the manufacturer’s protocols. The immunohistochemistry procedure was performed by the USC Immunohistochemistry Core. Images were acquired using Zeiss Axio Observer.A1 and Axio Imager.Z1 microscopes.

### RNA immunoprecipitation

The RIP assay was conducted using the Magna RIP Kit (Sigma Millipore, #17-701) according to the manufacturer’s protocol. Briefly, RIP lysate was collected from 10 cm dishes. Lysate was incubated with magnetic beads coupled with 5 µg of the antibody of interest (YTHDC2, YTHDF1–3) or corresponding mouse IgG or rabbit IgG overnight at 4ºC. After six washes and proteinase K digestion, RNA was eluted from the beads in 200 µl RLT buffer supplied in the RNeasy Micro Kit (Cat# 74004, Qiagen, Germany) for 2 min at room temperature. Magnetic separation rack was applied to pull beads to the side of the tube. Supernatant was collected to a new tube and combined with 400 µl of 100% ethanol. The mixture was transferred to an RNeasy MicroElute spin column (RNeasy Micro Kit) and centrifuged at >12 000 rpm at 4°C for 1 min. The column membrane was washed with 500 μl RPE buffer (RNeasy Micro Kit) once and with 500 µl of 80% ethanol once. The column was centrifuged at full speed for 5 min at 4°C to remove residual ethanol. For input samples, 1 µl of 14 µl eluted RNA was reverse transcribed. For RIP samples, 8 µl of 14 µl eluted RNA was reverse transcribed. Further analysis was performed using RT-qPCR using the primers in Supplementary Table S1.

Each RIP fraction was normalized to the input to account for RNA sample differences: ΔCt[normalized RIP] = (Ct[RIP] − log_2_(RIP dilution factor)) − (Ct[input] – log_2_(input dilution factor)), where RIP dilution factor = fraction of RIP used for cDNA synthesis or 8/14 and input dilution factor = fraction of input RNA saved × fraction of input RNA used for cDNA synthesis or 10/100 × 1/14. %Input for each RIP fraction was calculated as follows: %input = 2^(−ΔCt[normalized RIP])^. Enrichment was calculated relative to background antibody (mouse IgG or rabbit IgG) as follows: fold enrichment = 2(−ΔΔCt[RIP/IgG]), where ΔΔCt[RIP/IgG] = ΔCt[normalized RIP] − ΔCt[normalized IgG]. Finally, relative fold enrichment was calculated based on negative control transcript that does not bind the RIP protein using the following equation: relative fold enrichment = fold enrichment[transcript of interest]/fold enrichment[negative control transcript]. RIP efficiency was evaluated by western blot of the input RIP lysate and a fraction of the final RIP wash in probing for the RIP protein of interest.

### Clinical databases

TCGA analysis was performed using the TCGA PANCAN and TCGA BLCA datasets for all cancer types and for BC. SLC7A11 mRNA expression was assessed for an association with histologic grade, primary therapy outcome, overall survival, disease-specific survival and progression-free interval. Analysis was performed using the UCSC Xena exploration tool (45).

### Statistical analysis

*P*-values were calculated using two-tailed Student’s *t*-test between two groups. One-way ANOVA and two-way ANOVA were used to compare multiple groups. Data are presented as mean ± standard error of the mean from at least three independent experiments. Significance codes: ^****^P< 0.00001, ***P< 0.001, **P< 0.01 and *P< 0.05. GraphPad Prism version 9, Excel version 16.68 or R version 4.1.3 was used for statistical analysis.

## Results

### Cisplatin-sensitive and cisplatin-resistant cells have distinct m^6^A profiles

**Figure 1.**
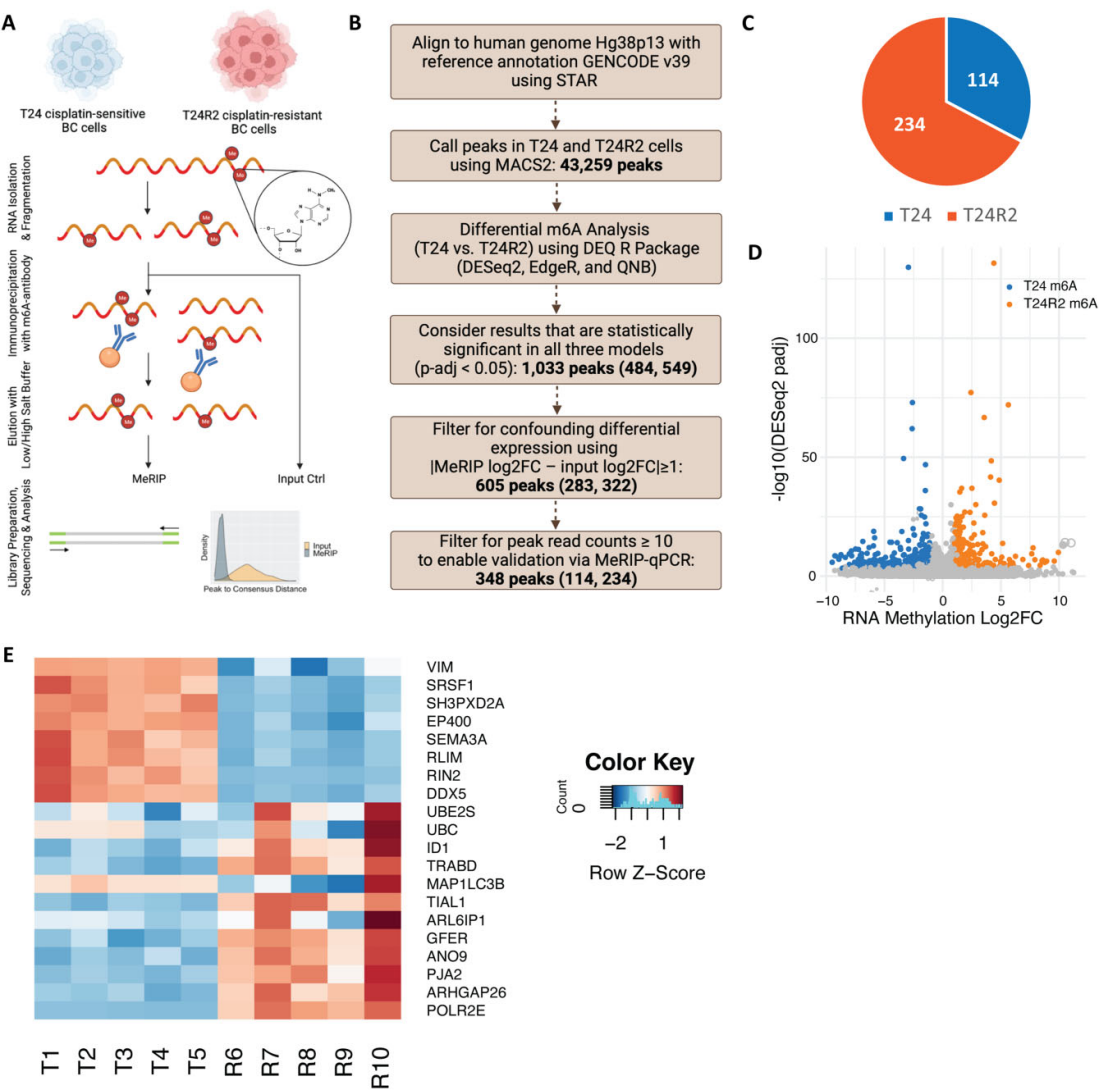
Cisplatin-sensitive and cisplatin-resistant cells have distinct m^6^A profiles. (**A**) MeRIP-seq bench discovery workflow and (**B**) bioinformatics pipeline to identify robust m^6^A peaks that are differentially methylated between T24 and T24R2. Numbers in parentheses refer to peaks hypermethylated in T24 and peaks hypermethylated in T24R2. (**C**) Summary of filtering results comparing T24 to T24R2, with 114 peaks hypermethylated in T24 and 234 peaks hypermethylated in T24R2. (**D**) Volcano plot of statistically significant differential MeRIP results (*P*< 0.05 by DESeq2, edgeR and QNB). T24 m^6^A represents the 114 peaks hypermethylated in T24 cells with log_2_FC < −1 and T24R2 m^6^A represents the 234 peaks hypermethylated in T24R2 cells with log_2_FC > 1. (**E**) Heatmap of top 20 differentially methylated transcripts ranked by DESeq2 *P*-adj.

We undertook a broad discovery approach comparing cisplatin-sensitive T24 BC cells (IC_50_: 10 µM cisplatin) to established cisplatin-resistant T24R2 BC cells (IC_50_: 100 µM cisplatin) (Supplementary Figure S1A). We used a low-input MeRIP-seq protocol (29) and a stringent differential analysis pipeline incorporating all recommended best practices published to date (39), including five replicates per condition and filtering criteria to mitigate the confounding effect of differential expression on differential methylation (Figure 1A and B). Without these critical additional steps, differential MeRIP-seq analysis can erroneously identify differentially methylated transcripts as a result of their underlying differential expression levels, as illustrated by the linear relationship between LFCs of RNA expression versus LFCs of RNA methylation (Supplementary Figure S1B). This is further illustrated when visualizing specific differentially methylated candidates (Supplementary Figure S1C and D). When the methylation amplitude is compared directly across all samples, the differences in m^6^A in this particular transcript between sensitive and resistant cells appear significant (Supplementary Figure S1C); however, when first normalizing the samples for underlying transcript expression levels, it becomes apparent that all samples contain methylation peaks in this transcript and that the difference in amplitude between sensitive and resistant cells is actually not significant for this particular peak (Supplementary Figure S1D).

As a first pass quality control measure, we applied principal component analysis to all 43 259 peaks called by MACS2 based on pull-down versus input in all 10 replicates (5 sensitive and 5 resistant). The sensitive and resistant replicates clustered tightly into two distinct groups, with PC1 and PC2 representing 82% and 3% variation, respectively, reflecting the power of the study design to accurately discriminate the two conditions (Supplementary Figure S1E). Encouraged by these preliminary results, we proceeded with differential methylation analysis using three statistical models (DESeq2, edgeR and QNB) to evaluate statistical significance (*P*< 0.05). We identified 1033 differentially methylated peaks between cisplatin-sensitive and cisplatin-resistant cells, of which 605 remained after filtering for differential expression and 348 remained after selecting those with at least 10 reads. Two hundred thirty-four of these peaks were hypermethylated in resistant cells, and 114 were hypermethylated in sensitive cells (Figure 1C). The volcano plot represents the 1033 statistically significant peaks, highlighting the 348 peaks that meet all the filtering criteria and are unique to either sensitive or resistant cells (Figure 1D). Peak distribution differed between sensitive and resistant cells, with an increase in 3′ untranslated region (3′UTR) peaks in T24R2 cells and increase in intronic peaks in T24 cells (Supplementary Figure S1F). Filtering out peaks with <10 reads removed primarily intronic peaks as those would be typically degraded quickly (Supplementary Figure S1G). The top 20 differentially methylated transcripts based on DESeq2 *P*-adj were visualized using a heatmap (Figure 1E).

### Filtering and validating candidate transcripts

**Figure 2.**
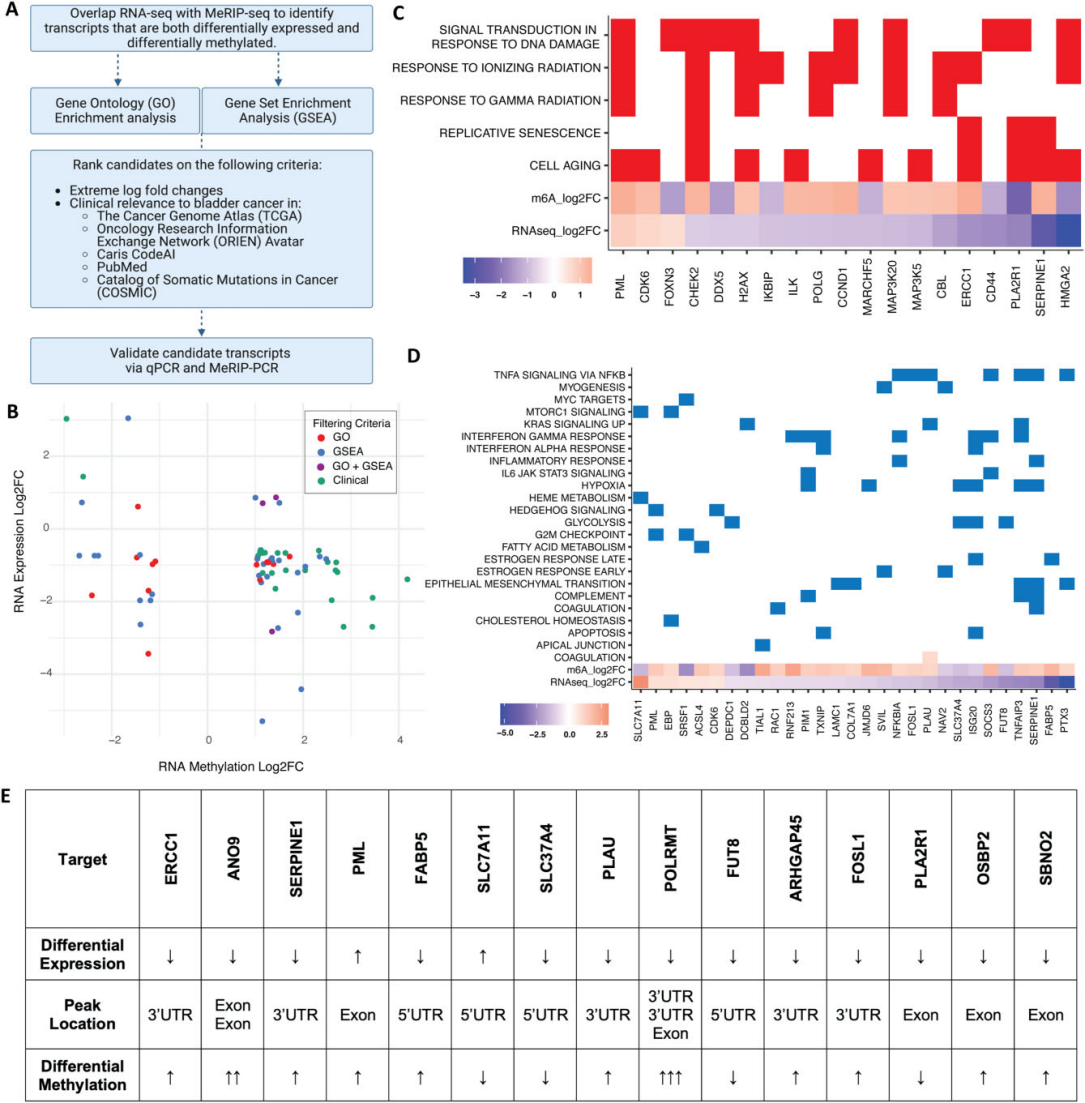
Identification of cancer-relevant transcripts that are both differentially methylated and differentially expressed in cisplatin-resistant cells. (**A**) Discovery and filtering pipeline. (**B**) RNA expression log_2_FC versus RNA methylation (m^6^A) log_2_FC of the 130 transcripts (162 peaks) that are both differentially methylated and differentially expressed between T24 and T24R2. Peaks are colored based on filtering criteria: GO, GSEA, both or neither. (**C**) Differentially methylated and expressed transcripts statistically significantly associated with GO terms (*P*-adj < 0.05). (**D**) Differentially methylated and expressed transcripts statistically significantly associated with GSEA cancer hallmarks (*P*-adj < 0.05). (**E**) Summary table of mRNA expression, methylation and m^6^A peak location for the top 15 candidate transcripts. Arrows indicate expression or methylation levels in T24R2 compared to T24. Each arrow indicates the directionality (increase or decrease) of changes in individual m^6^A peaks within the listed transcript corresponding to the order listed in the ‘Peak Location’ row.

**Figure 3.**
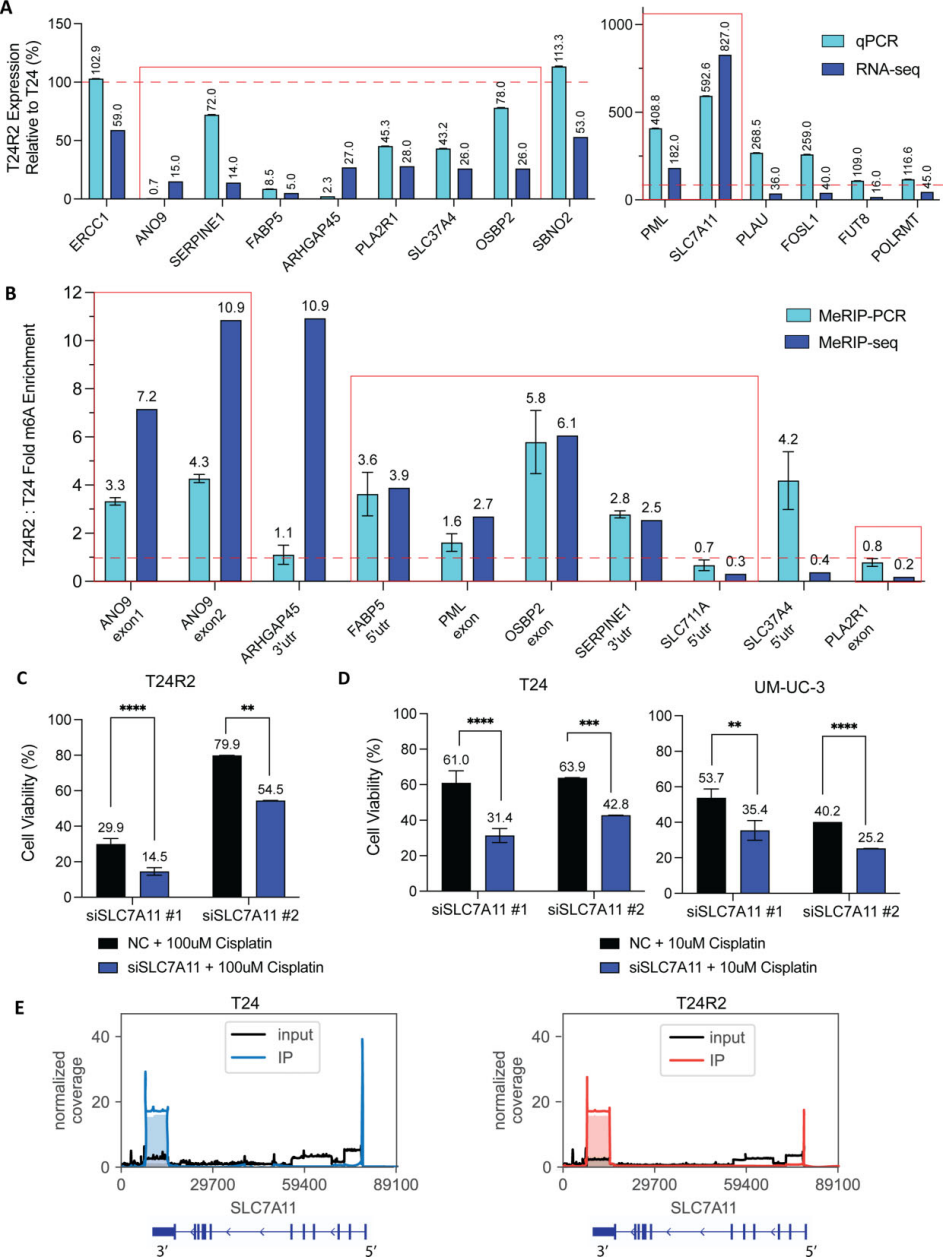
Validation of top 15 differentially methylated and expressed candidate transcripts. (**A**) Nine of 15 candidate transcripts (boxed) were validated as differentially expressed using qPCR, corroborating RNA-seq results. (**B**) Seven of the nine candidate transcripts (boxed) were validated by MeRIP-qPCR, corroborating MeRIP-seq results. Dotted lines in panels (A) and (B) represent the validation threshold. Functional validation of SLC7A11 evaluating its effect on cisplatin resistance via siSLC7A11 knockdown (two independent siRNAs) followed by cisplatin treatment in (**C**) T24R2 cells (CDI: 0.338, 0.681), (**D**) T24 cells (CDI: 0.515, 0.678) and UM-UC-3 cells (CDI: 0.661, 0.628). CDI < 0.7 indicates statistically significant synergy. Experiments were performed using three biological replicates. Significance codes: ^****^P< 0.00001, ***P< 0.001 and **P< 0.01. (**E**) m^6^A coverage plot for SLC7A11 in T24 and T24R2 cells with differential peak at the 5′UTR, and consistent (no difference) peak at the 3′UTR.

Since we hypothesized that m^6^A regulates the expression level of gene transcripts that promote transition to a drug-resistant state in BC, we overlapped the MeRIP-seq differential methylation results with RNA-seq differential expression from the same cisplatin-sensitive and cisplatin-resistant cell lines (Supplementary Figure S2A–C). We then applied the filtering pipeline outlined in Figure 2A in order to home in on transcripts relevant to cancer progression, as follows: We identified 130 candidate transcripts (162 out of 348 m^6^A peaks) that were both differentially expressed and differentially methylated (Figure 2B). We used GO and GSEA to filter the 130 transcripts based on membership in GSEA hallmarks of cancer pathways (Figure 2C) and relevance to GO terms like response to chemotherapy and radiation therapy (Figure 2D). We further ranked the resulting transcripts based on LFC and relevance to BC in the existing literature and clinical databases such as TCGA, the Oncology Research Information Exchange Network Avatar and the Catalog of Somatic Mutations in Cancer. After ranking, we selected the top 15 transcripts (Figure 2E) for *in vitro* validation by qPCR and by targeted immunoprecipitation and PCR (MeRIP-PCR). Nine of the 15 differentially expressed transcripts were downregulated in RNA-seq, and 7 of these 9 were also downregulated by qPCR (Figure 3A, left). Six of the 15 differentially expressed transcripts were upregulated in RNA-seq, and 2 of these were also upregulated by qPCR (Figure 3A, right). Of the nine transcripts whose differential expression was validated by qPCR, seven were also validated as differentially methylated by MeRIP-qPCR (Figure 3B). To determine whether any of these seven candidate genes were drivers of cisplatin resistance, each was siRNA-depleted in cell lines treated with cisplatin (Supplementary Table S5). One candidate, SLC7A21/xCT, was identified as a driver of cisplatin resistance in three BC cell lines, with siRNA knockdown exhibiting significant synergy with cisplatin compared to scrambled control (Figure 3C and D, and Supplementary S3A–C). MeRIP-seq of SLC7A11 mRNA revealed decreased m^6^A levels at the 5′UTR in cisplatin-resistant cells compared to cisplatin-sensitive ones and no difference in m^6^A levels at other regions, including one unchanged peak at the 3′UTR (Figure 3E).

### In cisplatin-resistant cells, hypomethylated SLC7A11 transcript has decreased binding of its reader YTHDF3, resulting in decreased degradation, increased SLC7A11 protein and reduced ferroptosis

**Figure 4.**
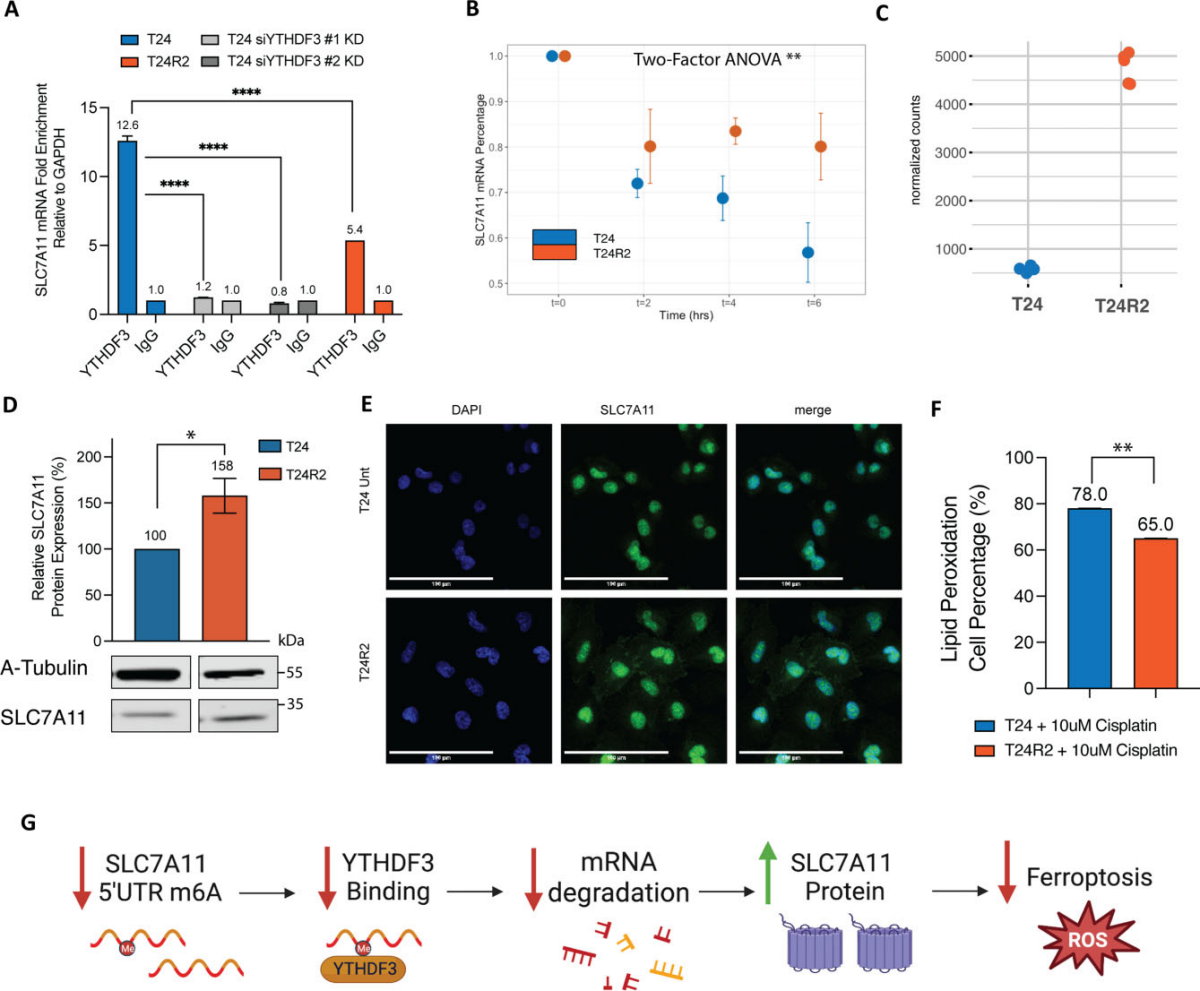
In cisplatin-resistant cells, hypomethylated SLC7A11 transcript has decreased binding of its reader YTHDF3 and decreased degradation, resulting in increased SLC7A11 protein and reduced ferroptosis. (**A**) SLC7A11 mRNA enrichment from YTHDF3 by RIP-qPCR, performed in T24 cells (two independent siYHDF3 knockdowns) and in T24R2 cells. Error bars reflect replicate measurements of SLC7A11 transcripts from the enriched protein. (**B**) SLC7A11 mRNA decay in T24 and T24R2 via actinomycin D RNA stability assay. (**C**) Normalized RNA-seq count of SLC7A11 in T24 and T24R2 cells. (**D**) Western blot and densitometric quantification, and (**E**) immunofluorescence of SLC7A11 protein in T24 and T24R2 cells with DAPI and SLC7A11. (**F**) Lipid peroxidation and ferroptosis flow cytometry assay using BODIPY 581/591 stain in T24 and T24R2 cells treated with cisplatin. (**G**) Model schema illustrating reduced SLC7A11 m^6^A, which leads to decreased YTHDF3 binding, increased mRNA stability and, in turn, increased SLC7A11 protein and decreased ferroptosis. Experiments were performed in three biological replicates. Significance codes: **P< 0.01 and *P< 0.05.

To identify an m^6^A reader associated with SLC7A11 transcript methylation, we performed RIP using antibodies specific for the most common m^6^A readers, YTHDF1–3 and YTHDC2, followed by qPCR for SLC7A11 (Figure 4A and Supplementary Figure S4A–C). We identified YTHDF3 as the corresponding reader for SLC7A11 m^6^A, with 12.6-fold relative enrichment of SLC7A11 mRNA upon YTHDF3 pull-down that was abrogated by prior YTHDF3 siRNA depletion. Consistent with this, hypomethylated SLC7A11 transcript in cisplatin-resistant T24R2 cells had reduced YTHDF3 binding, resulting in reduced SLC7A11 mRNA enrichment of only 5.4-fold (Figure 4A and Supplementary Figure S4B). We examined the RNA-seq data and found no significant differences in mRNA expression levels between the three YTHDF readers, suggesting that this was not a factor in the specificity of reader binding (Supplementary Figure S4D). Since YTHDF3 is an m^6^A reader known to promote mRNA degradation, we evaluated SLC7A11mRNA stability in cisplatin-sensitive versus cisplatin-resistant cells. To do so, we blocked transcription using actinomycin D and evaluated the rate of degradation of SLC7A11 mRNA. We found that SLC7A11 transcripts that are relatively hypomethylated in T24R2 cells degraded at a slower rate, whereas an unmethylated control transcript (*GAPDH*) was not affected (Figure 4B and Supplementary Figure S4E). Consistent with this, SLC7A11 mRNA was increased 8-fold by RNA-seq in cisplatin-resistant cells (Figure 4C), as was SLC7A11 protein level by western blot (Figure 4D and Supplementary Figure S4F). Correspondingly, more prominent cytoplasmic distribution of SLC7A11 can be observed in resistant cells on immunofluorescence microscopy (Figure 4E).

SLC7A11 serves as a suppressor of ferroptosis, an iron-dependent cell death program induced by excessive lipid peroxidation at cellular membranes (46–48). Therefore, we treated T24 versus T24R2 cells with cisplatin and quantified lipid peroxidation, which showed that cisplatin-resistant T24R2 cells had significantly less ferroptosis (Figure 4F). Taken together, cisplatin-resistant cells have decreased SLC7A11 m^6^A associated with decreased binding to the YTHDF3 m^6^A reader and decreased transcript degradation, resulting in increased mRNA and protein levels of SLC7A11, which attenuates ferroptosis-mediated cell death (Figure 4G).

### Short-term cisplatin treatment of BC cell lines reduces SLC7A11 transcript methylation, resulting in decreased YTHDF3 binding, decreased degradation and increased mRNA and protein levels

**Figure 5.**
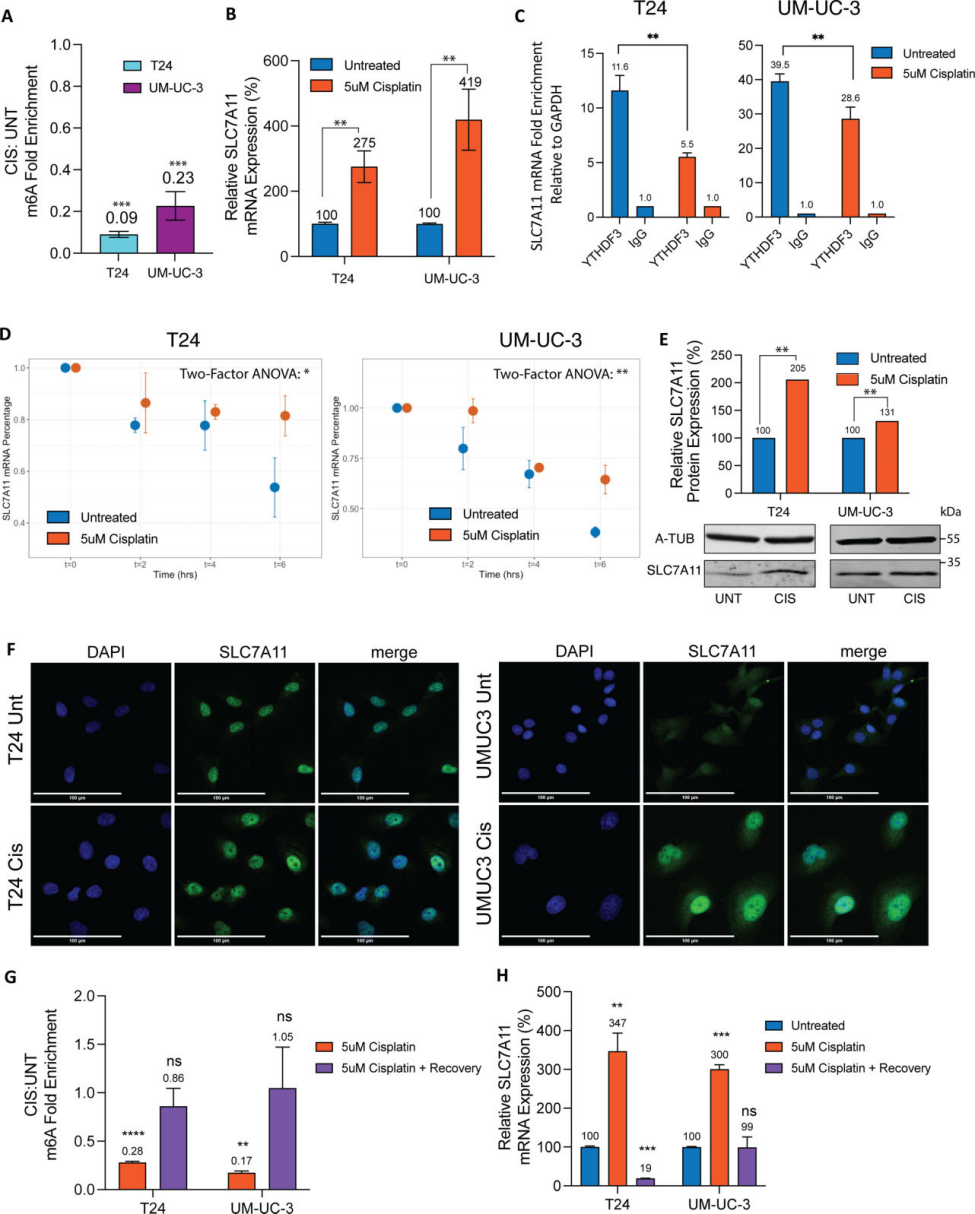
Short-term cisplatin treatment of cisplatin-sensitive BC cell lines reduces SLC7A11 transcript methylation, resulting in decreased YTHDF3 binding, decreased degradation and increased mRNA and protein levels. (**A**) *SLC7A11* m^6^A fold enrichment in cisplatin-treated cells relative to untreated cells, measured by MeRIP-qPCR in T24 and UM-UC-3 cells. (**B**) Relative SLC7A11 mRNA expression by qPCR of T24 and UM-UC-3 cells treated with cisplatin compared to untreated cells. (**C**) SLC7A11 mRNA enrichment from YTHDF3 by RIP-qPCR, performed in cisplatin-treated versus cisplatin-untreated T24 and UM-UC-3 cells. Error bars reflect replicate measurements of SLC7A11 transcripts from the enriched protein. (**D**) SLC7A11 mRNA decay in T24 and UM-UC-3 cells treated with cisplatin compared to untreated cells. (**E**) Western blot and densitometric quantification, and (**F**) immunofluorescence of SLC7A11 protein in T24 and UM-UC-3 cells treated with cisplatin compared to untreated cells, staining DAPI and SLC7A11. (**G**) SLC7A11 m^6^A fold enrichment in cisplatin-treated cells relative to untreated cells, measured by MeRIP-qPCR in T24 and UM-UC-3 cells after cisplatin treatment, and again after recovery from cisplatin treatment. (**H**) Relative SLC7A11 mRNA expression measured by qPCR in T24 and UM-UC-3 cells after cisplatin treatment, and again after recovery from cisplatin treatment. Experiments were performed in three biological replicates. Significance codes: ^****^P< 0.0001, ***P< 0.001, **P< 0.01 and *P< 0.05.

Having observed the role of SLC7A11 transcript methylation in established cisplatin-resistant cell lines, we evaluated whether these effects can be induced with short-term treatment of cisplatin-sensitive cells. We started by evaluating the expression of SLC7A11 mRNA in T24 and UM-UC-3 BC cell lines upon treatment with different concentrations of cisplatin over time and found a significant increase in SLC7A11 as early as 48 h in T24 cells and 72 h in UM-UC-3 cells, using both 5 and 10 µM cisplatin (Supplementary Figure S5A). We selected the earliest time point and lowest cisplatin concentration for each cell line to evaluate SLC7A11 m^6^A, mRNA and protein expression changes upon cisplatin treatment (5 µM for 48 h for T24 and 5 µM for 72 h for UM-UC-3). SLC7A11 m^6^A measured by MeRIP-qPCR was 90% and 80% depleted in T24 and UM-UC-3 cells, respectively, after cisplatin treatment (Figure 5A and Supplementary Figure S5B). This m^6^A decrease was associated with increase in SLC7A11 mRNA in both cell lines (Figure 5B). Consistent with these findings, YTHDF3 binding was significantly decreased in cisplatin-treated cells (Figure 5C), which was associated with reduced degradation and greater SLC7A11mRNA stability compared to an unmethylated control transcript (*GAPDH*) (Figure 5D and Supplementary Figure S5E) and increased SLC7A11 protein levels by western blot (Figure 5E). Correspondingly, more prominent cytoplasmic distribution of SLC7A11 can be observed in resistant cells in cisplatin-treated cells compared to untreated on immunofluorescence microscopy (Figure 5F). Hence, SLC7A11 transcript methylation phenotypes displayed by established cisplatin-resistant cells were recapitulated in cisplatin-sensitive cells as early as 48 h after cisplatin treatment, suggesting that m^6^A RNA modifications may regulate early changes that contribute to cisplatin resistance. We next asked whether the new pro-survival methylation profile is ‘remembered’ by cells and hypothesized that SLC7A11 transcripts may no longer retain the hypomethylated state once the chemotherapeutic stressor is removed. To test this hypothesis, we treated T24 and UM-UC-3 cells with short-term cisplatin, and then discontinued treatment and reanalyzed SLC7A11 mRNA methylation and expression when the cells fully recovered their proliferation after 2–3 weeks. Surviving cells indeed returned to their baseline phenotype with increased SLC7A11 m^6^A and corresponding reduced expression of SLC7A11 (Figure 5G and H, and Supplementary Figure S5F).

### Epitranscriptomic regulation and cancer promoting role of SLC7A11 are recapitulated in PDOs and clinical outcomes

**Figure 6.**
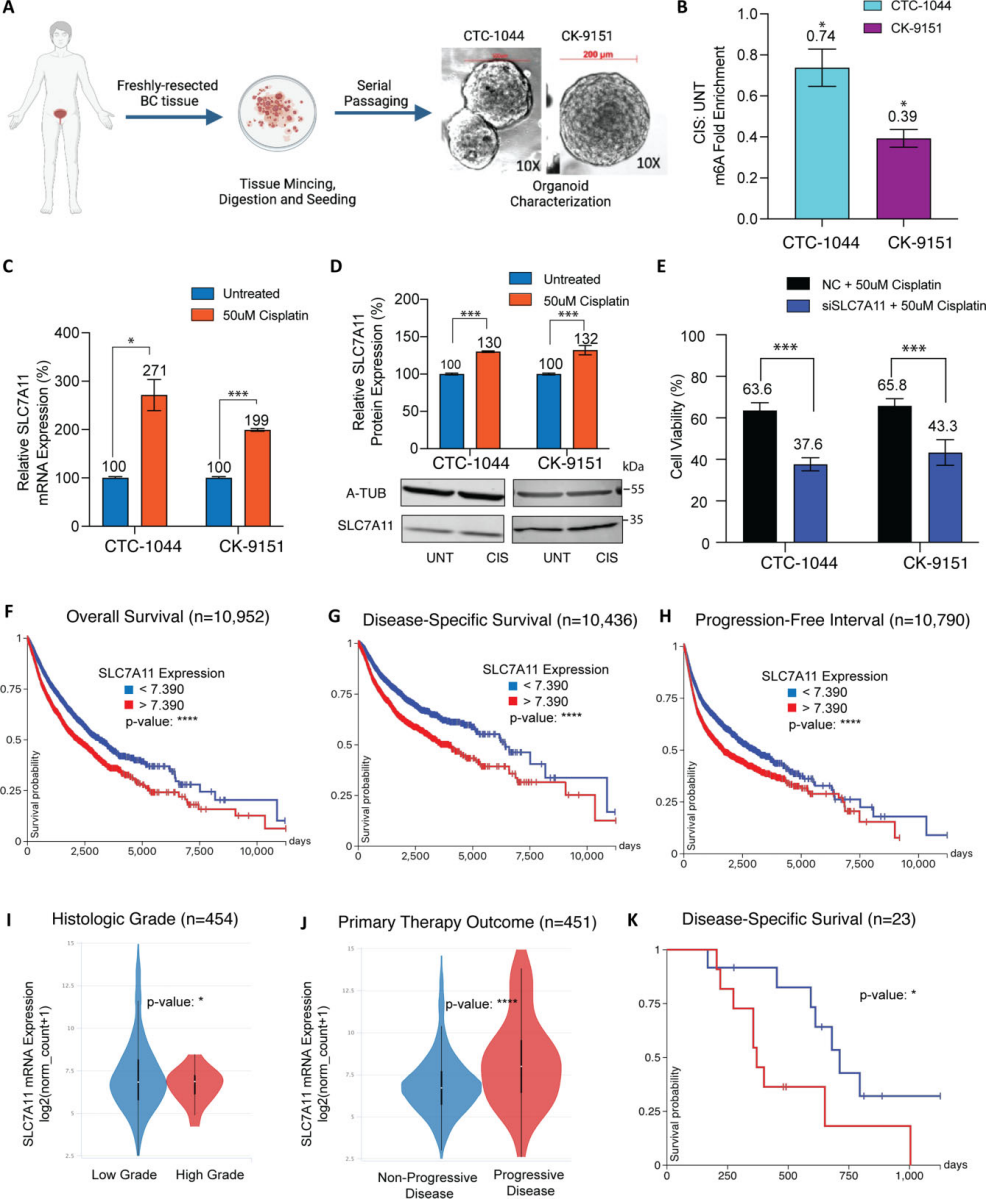
Epitranscriptomic regulation and cancer promoting role of SLC7A11 are recapitulated in PDOs and clinical outcomes. (**A**) Schema for establishing BC PDOs and representative bright-field images of CTC-1044 and CK-9151 at 10× magnification. (**B**) SLC7A11 m^6^A fold enrichment in cisplatin-treated cells relative to untreated cells, measured by MeRIP-qPCR in CTC-1044 and CK-9151. (**C**) Relative SLC7A11 mRNA expression by qPCR of CTC-1044 and CK-9151 PDOs treated with cisplatin, compared to untreated cells. (**D**) Western blot and densitometric quantification of SLC7A11 protein in CTC-1044 and CK-9151 PDOs treated with cisplatin compared to untreated. (**E**) Functional validation of SLC7A11 evaluating its effect on cisplatin resistance in PDOs via siSLC7A11 knockdown followed by cisplatin treatment in CTC-1044 (CDI: 0.5431747) and CK-9151 (CDI: 0.5660335). Kaplan–Meier curves for overall survival (**F**), disease-specific survival (**G**) and progression-free interval (**H**) in patients with all cancer types from TCGA, with high versus low SLC7A11 expression split at the median. SLC7A11 expression in BC patients with high versus low histologic grade (**I**) and non-progressive versus progressive disease after primary therapy (**J**) in TCGA. (**K**) Disease-specific survival in BC patients with progressive disease on primary therapy in TCGA, with high versus low SLC7A11 expression between highest and lowest quartiles. Experiments were performed in three biological replicates. Significance codes: ^****^P < 0.00001, ***P < 0.001 and *P < 0.05.

To validate the cell line findings in tumor tissues from BC patients, we generated and characterized PDOs from patient bladder tumor samples from freshly resected TURBTs (Figure 6A and Supplementary Figure S6A). For the experiments showed, we used one PDO generated in our lab (CTC-1044) and one obtained from the NCI repository (CK-9151). After 48 h of cisplatin treatment, m^6^A was significantly depleted (Figure 6B and Supplementary Figure S6B), which was associated with significantly increased levels of SLC7A11 mRNA and protein levels (Figure 6C and D). Consistent with these findings, siRNA-mediated depletion of SLC7A11 sensitized the PDOs to cisplatin (Figure 6E and Supplementary Figure S6C–E).

To further evaluate the clinical significance of our findings in larger cohorts of patients, we analyzed TCGA public database to evaluate associations between SLC7A11 expression and cancer-relevant clinical variables and outcomes. In TCGA, high expression of SLC7A11 was associated with lower overall survival, disease-specific survival and progression-free interval across all malignancies (Figure 6F–H). In BC specifically, patients with high histologic grade had higher SLC7A11 mRNA expression compared to those with low histologic grade (Figure 6I). Moreover, patients with progressive disease had higher SLC7A11 mRNA expression compared to patients with no progression (Figure 6J). Among patients with progressive disease, those with higher SLC7A11 mRNA expression had lower disease-specific survival (Figure 6K).

## Discussion

A major obstacle to achieving lasting cures for advanced malignancies is the emergence of acquired resistance to treatment, not only due to genetically resistant clones but also through phenotypic plasticity. In this study, we aimed to characterize the role of m^6^A RNA modifications in regulating the expression of genes that promote transition to cisplatin resistance in BC. m^6^A modifications play a dynamic role in cell fate and pluripotency, raising the possibility that this epitranscriptomic mechanism contributes to phenotypic plasticity and drug resistance in BC (9,49,50). It has been proposed that while most m^6^A sites are largely hard-wired, a smaller more variable subset of sites may serve as master regulators of specific cellular pathways (18). Writers and erasers are responsible for depositing and removing m^6^A, and readers in particular serve as effectors of downstream events, including alternative splicing, translation and degradation, thereby regulating cellular phenotypes and impacting cancer progression (10). Despite this multiplicity of observed regulatory mechanisms, the dominant function of m^6^A is to regulate RNA stability, leading to increases or decreases in gene expression (18).

In cancer, m^6^A has been dubbed a ‘double-edged sword’—the presence of modification at one site or its absence at another may contribute to cancer progression (3). It is thus critical to take an agnostic approach within each cancer of interest to accurately discover and identify transcripts associated with differential methylation and differential expression. To date, most studies have focused on a specific m^6^A effector (writer, eraser, reader) and considered transcriptome-wide m^6^A changes induced by knockout or overexpression of the effector of interest (26,51–53). These studies have significantly advanced the field. At the same time, effectors have a multitude of partners and targets. Thus, any specific m^6^A modifications or cancer phenotypes induced by effector manipulation are understood to occur in the context of numerous other transcripts and pathways potentially perturbed by that same effector manipulation. In this study, we took a different approach by directly mapping the epitranscriptomic landscape of chemotherapy-sensitive versus chemotherapy-resistant cells, thereby focusing our discovery on m^6^A alterations associated with these disease states rather than on specific effectors.

Using a well-established cell line model of cisplatin resistance in BC (28), we analyzed transcriptome-wide changes in m^6^A RNA modifications and gene expression using MeRIP-seq and RNA-seq. To do so, we adopted a rigorous informatics approach: taking into consideration the limitations of MeRIP-seq, implementing the guidelines for best practices outlined in (39) and using five tightly clustered replicates per condition. As a result, this study is, to our knowledge, the most well-powered differential MeRIP-seq analysis of its kind to date, as reflected by the high validation rate (78%) of MeRIP-seq targets by repeat targeted MeRIP-qPCR (Figure 3). In all, we identified 130 transcripts that were both differentially methylated and differentially expressed. It is possible that many of these candidates are involved, singly or in combination, to varying degrees in the transition to cisplatin resistance. However, here we sought to focus on a candidate driver of resistance with readily interpretable downstream mechanism of action as a proof of concept for the discovery approach used in this study. Having demonstrated the feasibility of this approach, it is our hope that it will be applied to additional transcript candidates and in other disease models.

Using this strategy, we identified SLC7A11/xCT to be differentially hypomethylated in resistant cells, a modification that decreased its binding to the reader YTHDF3. Reduced association with YTHDF3 in turn decreased SLC7A11 transcript degradation, leading to increased levels at the mRNA and protein levels, reduced ferroptosis and enhanced survival. Notably, these changes were apparent not only in established cisplatin-resistant cells, but also in cisplatin-sensitive cell lines and PDOs after brief (48 h) treatment with cisplatin. Upon removal of chemotherapeutic stressor, surviving cells eventually resumed proliferating and returned to their baseline phenotype with high SLC7A11 m^6^A and low SLC7A11 expression, underscoring the rapid adaptive nature of phenotypic plasticity.

SLC7A11 is a 1:1 cystine–glutamate antiporter that replenishes cystine, a key precursor of glutathione biosynthesis and antioxidant defense (46). SLC7A11 is overexpressed in multiple human cancers and promotes tumor growth by suppressing ferroptosis, a form of iron-dependent regulated cell death induced by excessive lipid peroxidation at cellular membranes (46–48). SLC7A11 was first linked to cisplatin resistance in ovarian cancer and has since been studied in multiple other cancer types (54). SLC7A11 inhibitors such as sulfasalazine and sorafenib, as well as other ferroptosis inducers, are currently being tested in combination therapy with cisplatin and doxorubicin in clinical trials for head and neck carcinomas, acute myeloid leukemia, glioblastoma, ovarian cancer, hepatocellular carcinoma and renal cell carcinoma (47,55). In BC, we are aware of two studies involving SLC7A11 (48,56). Both found an association between SLC7A11 overexpression and poor clinical outcome, and one reported a link with cisplatin resistance (56). Although these prior reports in BC did not address epitranscriptomic regulation, this mechanism was observed in other malignancies. In lung adenocarcinoma, one study reported that the YTHDC2 m^6^A reader binds an SLC7A11 m^6^A site at the 3′UTR, leading to decreased mRNA stability and expression (57). Another study in hepatoblastoma reported that m^6^A-dependent inhibition of SLC7A11 deadenylation promotes tumorigenesis (58). In our current study, *in silico* analysis of publicly available databases confirmed that SLC7A11 levels are associated with clinical outcomes in BC and other malignancies. While databases such as TCGA are not annotated in a manner that can directly inform our questions about cisplatin resistance and epitranscriptomic regulation, they nonetheless attest to the potential clinical relevance of these targets. Future studies comparing large cohorts of tumor samples from TURBTs (pre-cisplatin) versus tumor samples from radical cystectomies (post-cisplatin) could further expand on the clinical relevance of our mechanistic findings.

One potential limitation of MeRIP-seq in this setting is its 100 nt resolution for determining exact methylation site. While we demonstrate a statistically significant association between this m^6^A site and SLC7A11 transcript enrichment on YTHDF3 (Figure 4A), we cannot rule out effector interactions at additional sites on SLC7A11, nor the possibility that cisplatin induces global m^6^A changes, with some targets losing or gaining more m^6^A than others. Although an important consideration, the bidirectionality of statistically significant m^6^A changes identified (some sites hypermethylated and others hypomethylated) makes it less likely that global methylation changes drive these findings. Future studies could further elucidate these m^6^A profiles using newly developed tools with single-nucleotide resolution, such as GLORI (59). Another limitation of MeRIP-seq is its inability to distinguish definitively between m^6^A and *N*^6^,2′-*O*-dimethyladenosine (m^6^Am). We conducted an adjunct experiment (Supplementary Figure S3D) individually knocking down ALKBH5 (m^6^A eraser) and FTO (primarily m^6^Am eraser), and we found that only ALKBH5 knockdown altered SLC7A11 expression. This suggests that the differentially methylated site on SLC7A11 is more likely to be m^6^A rather than m^6^Am, although it does not entirely rule out the possibility that m^6^Am plays a role as well. Additional genetic manipulations of various effectors and specific m^6^A sites could offer further insights about their potential roles in regulating specific methylation sites associated with transcript degradation and adaptive chemoresistance. For example, m^6^A readers are generally recognized to be promiscuous, interacting with a broad array of targets; therefore, additional functional interaction of SLC7A11 with other readers cannot be ruled out in this model. It is also important to note that while YTHDF readers are predominantly implicated in RNA stability and degradation, m^6^A at 5′UTR has also been associated with changes in translation through direct interaction with translation factor eIF3 (60). It is possible that changes in translation could be at play in addition to YTHDF3-mediated mRNA degradation; however, teasing out the relative contribution of mRNA degradation, eIF3-mediated translation and other mechanisms of translation (elongation, termination, ribosome recycling) would be technically challenging.

Our current study demonstrates that SLC7A11 mRNA methylation and degradation are reduced in response to cisplatin treatment, contributing to a rapid increase in SLC7A11 antiporter level that mitigates ferroptosis and promotes survival of BC cells. These findings highlight epitranscriptomic regulation as an important contributor to phenotypic plasticity in cancer cells. This capacity for rapid adaptation to chemotherapeutic stress may serve as an initial survival tactic that ‘buys time’ until clonal selection of adaptive mutations can occur. Therefore, targeting such early adaptive mechanisms may be an effective strategy to derail the transition to therapy resistance before it is firmly established.

## Supplementary Material

zcad054_Supplemental_File

## Data Availability

The sequencing data have been deposited in the Gene Expression Omnibus database (https://www.ncbi.nlm.nih.gov/geo/) and can be accessed by GSE231836. All other relevant data are within the paper.
