# Supplementary material for: m6A epitranscriptome analysis reveals differentially methylated transcripts that drive early chemoresistance in bladder cancer
Source: NAR Cancer. 2023 Nov 16;5(4):zcad054. doi: 10.1093/narcan/zcad054 (PMC10653028; doi:10.1093/narcan/zcad054)
Supplement: zcad054_Supplemental_File [file zcad054_supplemental_file.pdf]

SUPPLEMENTARY TABLES & FIGURES

Supplementary Table 1. RT-qPCR and MeRIP-qPCR Primers

**RT-qPCR Primers**	**FWD**	**REV**
GAPDH	TCA AGG CTG AGA ACG GGA AG	GGA CTC CAC GAC GTA CTC AG
SETD7	GGGGTTCAGAGACCTGGAAT	GCATGGTGAGAGGATGTGAC
CLUC	GCTTCAACATCACCGTCATTG	CACAGAGGCCAGAGATCATTC
GLUC	CGACATTCCTGAGATTCCTGG	TTGAGCAGGTCAGAACACTG
METTL3	CCA CTG ATG CTG TGT CCA TC	GGA GAC CTC GCT TTA CCT CA
FTO	ACT TGG CTC CCT TAT CTG ACC	TGT GCA GTG TGA GAA AGG CTT
ALKBH5	TTC TCT TCC TTG TCC ATC TC	ATC CTC AGG AAG ACA AGA TTA G
YTHDC2	AGA TAA GCA GGG ATG GGC AG	AAG ACG TTC CCA TAA CTG GAG C
YTHDF1	GCA CAC AAC CTC CAT CTT CG	AAC TGG TTC GCC CTC ATT GT
YTHDF2	ACT TGA GTC CAC AGG CAA GG	AAG CAG CTT CAC CCA AAG AA
YTHDF3	TGA CAA CAA ACC GGT TAC CA	TGT TTC TAT TTC TCT CCC TAC GC
SLC7A11	GCG TGG GCA TGT CTC TGA C	GCT GGT AAT GGA CCA AAG ACT TC
ANO9	CTC CGA GCA GTG GGA CTA TG	AGT GCG GTA CAG GCC AAA G
ERCC1	CCT TAT TCC GAT CTA CAC AGA GC	TAT TCG GCG TAG GTC TGA GGG
SERPINE1	GCA CCA CAG ACG CGA TCT T	ACC TCT GAA AAG TCC ACT TGC
PML	CGC CCT GGA TAA CGT CTT TTT	CTC GCA CTC AAA GCA CCA GA
FABP5	TGA AGG AGC TAG GAG TGG GAA	TGC ACC ATC TGT AAA GTT GCA G
SLC37A4	AGG TAG CTC CTA CAT GAG TGC	GGG TTC CCG TAG TTG GAC AG
PLAU	GCT TGT CCA AGA GTG CAT GGT	CAG GGC TGG TTC TCG ATG G
POLRMT	CGC CAC ATC CAC CCT GTT C	GGA CCA TCG AAA GGT GTC TGG
FUT8	AAC TGG TTC AGC GGA GAA TAA C	TGA GAT TCC AAG ATG AGT GTT CG
ARHGAP45	CTC CTG TCC ATC TAC TCG CTG	CCT GCG CTT CTC GTG TTC A

FOSL1	CAG GCG GAG ACT GAC AAA CTG	TCC TTC CGG GAT TTT GCA GAT
PLA2R1	TGG AGT GGC AGG ATA AAG GAA	AGG GTC AGA ACC GAT TTA CCT
OSBP2	GAA CCT GTG TCC GAG ACG AC	CCT GAG CTT GAC TCT GAC CC
SBNO2	ACT CCC TGT CGG ACA TCG T	GAA CAG CTT ATC GTG GGT GGA
**MeRIP-qPCR Primers**	**FWD**	**REV**
SLC7A11 5'UTR m^6A region	CAG CGC TAT AGT GTT CAC AGG T	AGT AGT AAT TAG ATC GCT GTG AAG G
ANO9 Exon m^6A region 1	AGC AAA GAG TCC CGA GGA GA	ACA GTC CTT CGT CGG TTG TT
ANO9 Exon m^6A region 2	CGT TGA TCC TGG GGA GGA AGG	GTC TTT CCT ACC CGC TTC TGT C
SERPINE1 3'UTR m^6A region	GCC CCT CTT TTT CCC CTT GAT	ACT CCG TCC TTT TGA TCC CC
PML Exon m^6A region	GCC AGG TGG TAG CTC ACG	ACT GGC CAT CTC CTC GTA GT
FABP5 5'UTR m^6A region	CTT TCC CTC CCT GTC GCA TC	GAT TTC TGC GGG AAA CTG CG
SLC37A4 5'UTR m^6A region	CTC CCT TTA TAG CCG CCT TCT	CAG TTT GGC GCT CAG TAA TCT C
ARHGAP45 3'UTR m^6A region	GTA CAC AGT GGG GTC TCT CG	GCC ACA GAA AAC ACC CGA TT
PLA2R1 Exon m^6A region	CCC CCG ACA ATA GTC TGT CAT	CCC CAC CTA TGG AGG TAT GT
OSBP2 Exon m^6A region	TAA CGG CAC CTG AGG AGC AT	TCC CCA GGT TGC TGA TCC AT

Supplementary Table 2. siRNA References & Conditions

Target	Company	Catalog#	Concentration	Time
Negative Ctrl	IDT	51-01-14-03	10nM	24-48HRS
SLC7A11	IDT	hs.Ri.SLC7A11.13.2<br>hs.Ri.SLC7A11.13.3	20nM<br>20nM	24HRS<br>24HRS
ANO9	SCBT	sc-96721	20nM	24HRS
SERPINE1	IDT	hs.Ri.SERPINE1.13.2	10nM	24HRS
PML	SCBT	sc-36284	20nM	48HRS
FABP5	IDT	hs.Ri.FABP5.13.2	10nM	24HRS

PLA2R1	IDT	hs.Ri.PLA2R1.13.5	10nM	24HRS
OSBP2	IDT	hs.Ri.OSBP2.13.1<br>hs.Ri.OSBP2.13.3	20nM/each	48HRS
YTHDF3	IDT	hs.Ri.YTHDF3.13.1<br>hs.Ri.YTHDF3.13.3	10nM<br>20nM	48HRS<br>48HRS

Supplementary Table 3. Western Blot Antibodies

**Protein Target**	**Size (kDa)**	**Dilution**	**Company**	**Catalog#**	**Lot#**
GAPDH (Mouse)	37	1:5000	Invitrogen	437000	XD350279
β-ACTIN (Mouse)	45	1:5000	Cell Signaling Technologies	8H10D10 #3700S	Lot 20
α-TUBULIN (Mouse)	55	1:4000	Invitrogen	62204	2407525
SLC7A11 mAb for WB (Rabbit)	35	1:1000	Cell Signaling Technologies	D2M7A #12691	Lot 5
YTHDC2 (Rabbit)	160	1:1000	Abcam	ab176846	GR25634746
YTHDF1 (Rabbit)	60	1:4000	Proteintech	17479-1-AP	00105213
YTHDF2 (Rabbit)	62	1:1000	Proteintech	24744-1-AP	00110531
YTHDF3 (Mouse)	64	1:500	SCBT	sc-377119	A0422
Goat anti-Rabbit IgG Secondary Antibody	-	1:10,000	Invitrogen	SA535571	VA296084
Goat anti-Mouse IgG Secondary Antibody	-	1:10,000	LI-COR	926-68070	D20316-15

Supplementary Table 4. Immunofluorescence Antibodies

**Protein Target**	**Size (kDa)**	**Dilution**	**Company**	**Catalog#**	**Lot#**
SLC7A11 pAb for IF (Rabbit)	-	1:500<br>1:1000	Invitrogen	PA1-16[illegible]93	XJ3707839
Goat anti-Rabbit Alexa Fluor 488	-	1:1000	Invitrogen	A-11008	2420731

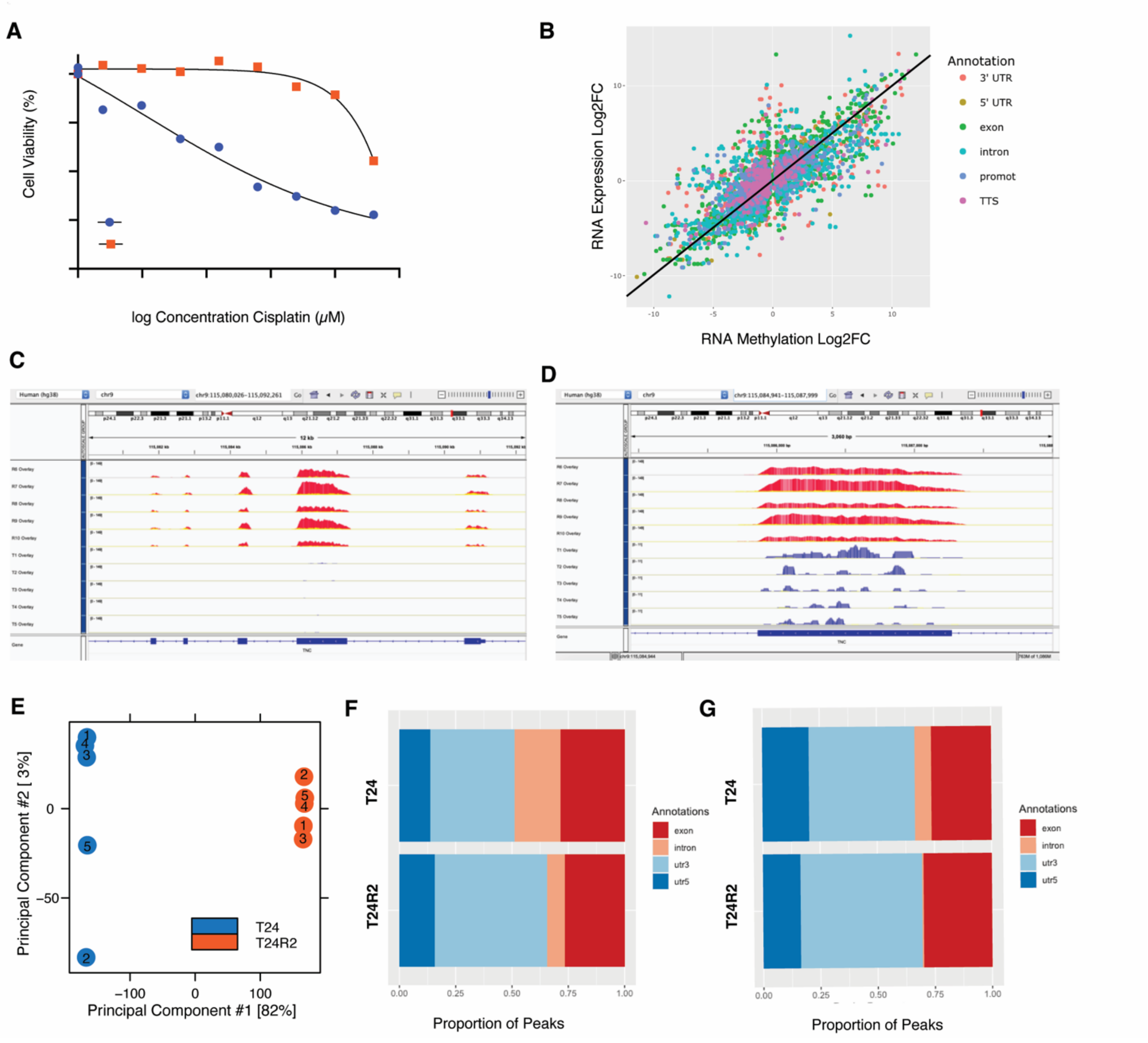

Supplementary Figure 1. Differential MeRIP-seq optimization and annotation between T24 and T24R2 BC cells. (A) Dose-response curve for cisplatin treatment in T24 and T24R2 cells. (B) Plotting raw RNA expression Log2FC vs RNA Methylation Log2FC demonstrates a pronounced y=x (black) linear relationship, underscoring the critical need to filter for differential expression which would otherwise confound differential methylation calling. Representative IGV plot of m^{6}A peak found on y=x line in (B) scaling all samples (C) together or by condition (D). (E) PCA plot of MeRIP samples with 5 replicates per cell line. Peak Annotation before (F, 605 peaks) and after (G, 348 peaks) removing peaks with fewer than 10 reads, which results in loss of primarily intronic differential peak calls.

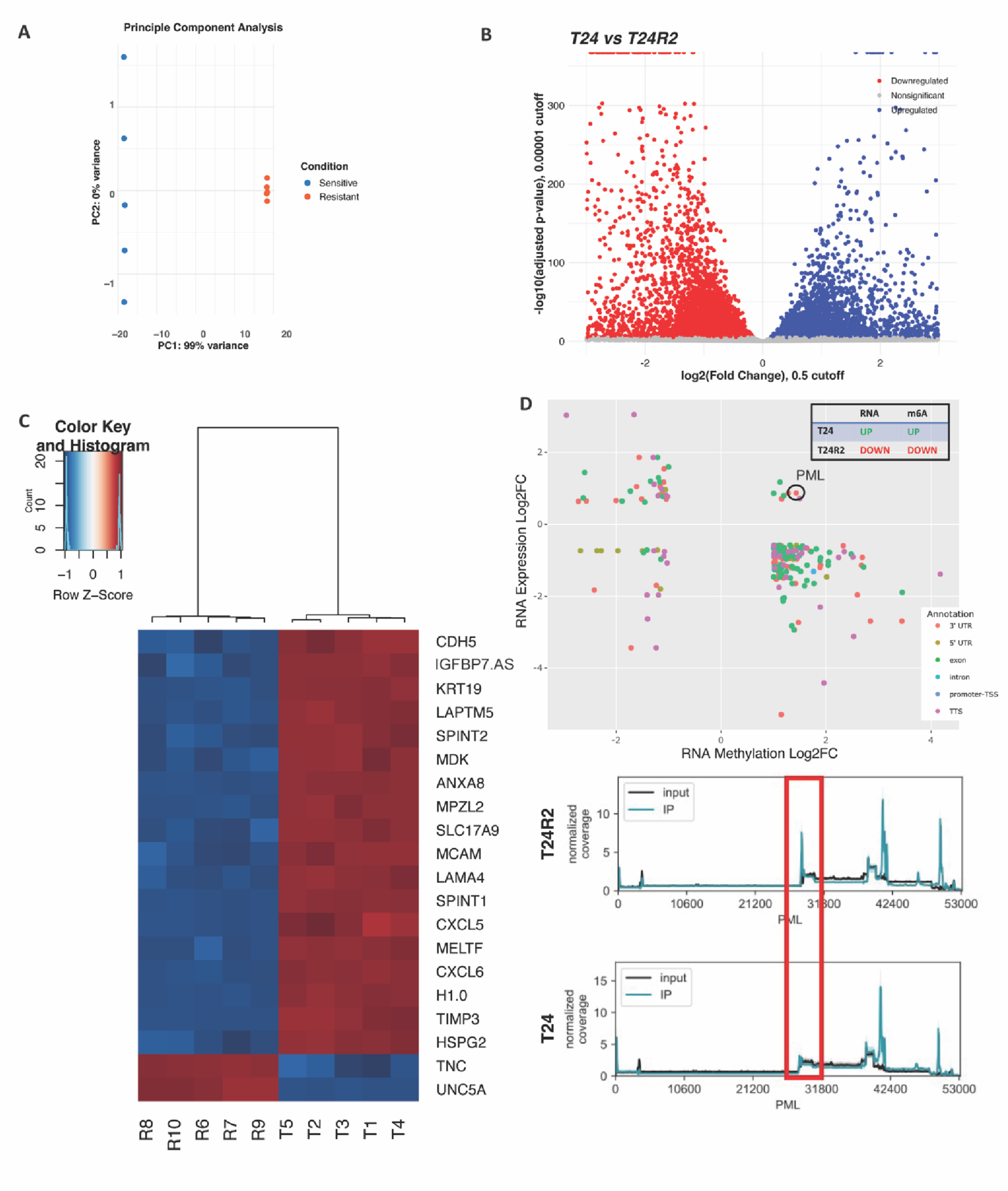

Supplementary Figure 2. Differential RNA-seq between T24 and T24R2 BC cells. (A) PCA plot of RNA-seq samples with 5 replicates per cell line. (B) Volcano Plot of statistically significant differential RNA-seq results ($p<0.00001$). Downregulated represents the peaks with log2FC < -0.5 and Upregulated with log2FC > 0.5. (C) Heatmap of top 20 differentially expressed transcripts ranked by log2FC. (D) RNA expression Log2FC vs RNA Methylation Log2FC after filtering for confounding differential expression and m^6A coverage plot for PML, a representative transcript that has increased expression and methylation in T24R2 compared to T24.

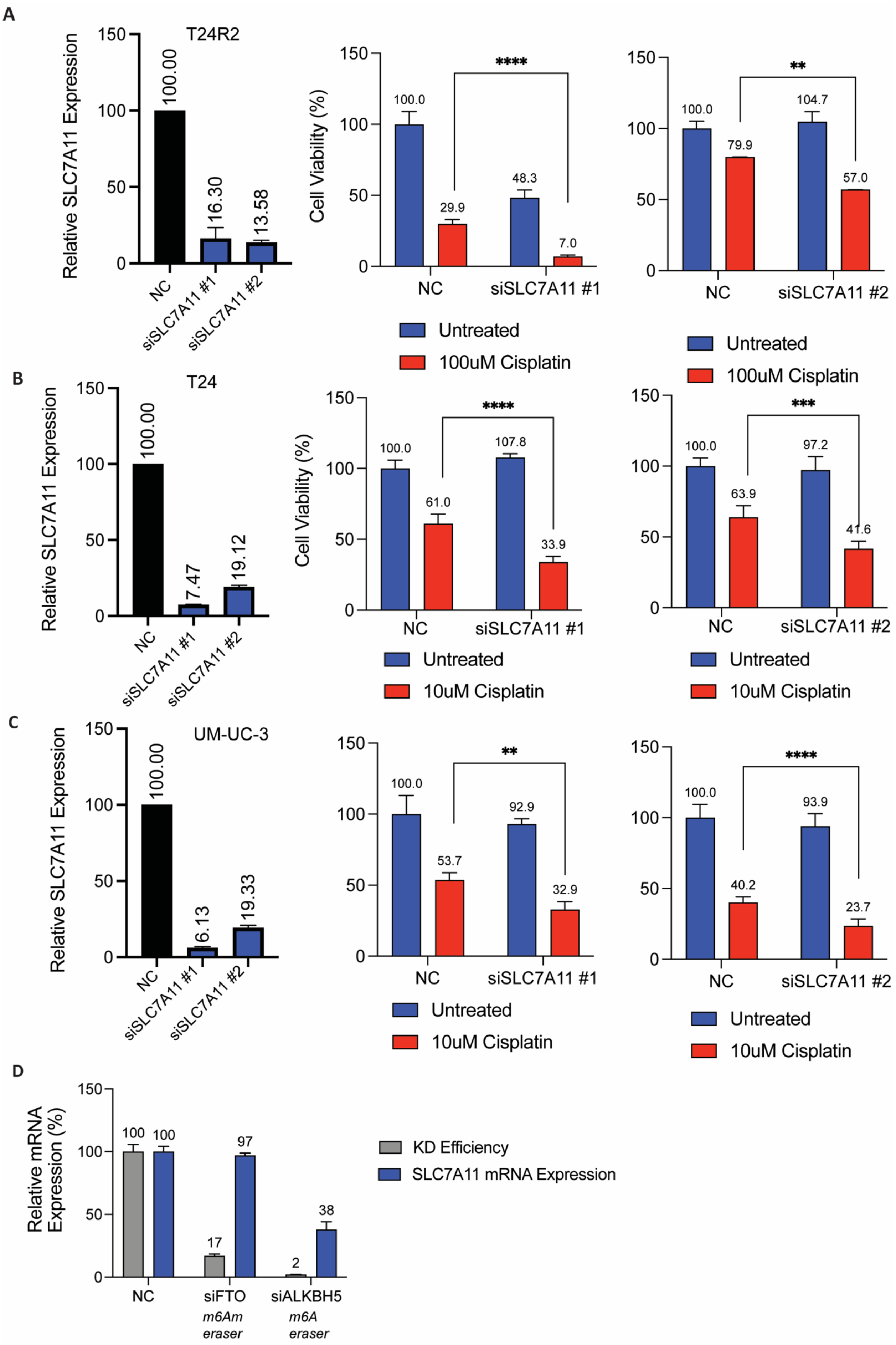
A
T24R2
Relative SLC7A11 Expression
100.00
16.30
13.58
NC
siSLC7A11 #1
siSLC7A11 #2
Cell Viability (%)
****
100.0
29.9
48.3
7.0
**
100.0
79.9
104.7
57.0
Untreated
100uM Cisplatin
B
T24
100.00
7.47
19.12
****
100.0
61.0
107.8
33.9
***
100.0
63.9
97.2
41.6
10uM Cisplatin
C
UM-UC-3
100.00
6.13
19.33
**
100.0
53.7
92.9
32.9
****
100.0
40.2
93.9
23.7
D
Relative mRNA Expression (%)
100
100
17
97
2
38
siFTO
m6Am eraser
siALKBH5
m6A eraser
KD Efficiency
SLC7A11 mRNA Expression

Supplementary Figure 3. siRNA-knockdown efficiency and cisplatin viability in BC cells. SLC7A11 siRNA-knockdown efficiency and cell viability upon siSLC7A11 knockdown (two independent siRNAs) with and without cisplatin treatment in (A) T24R2, (B) T24, (C) UM-UC-3 cells. NC= negative control (scrambled siRNA). (D) ALKBH5 and FTO siRNA knockdown efficiency and SLC7A11 mRNA expression upon respective knockdown. Significant codes: '****': $p < 0.00001$, '**': $p < 0.01$.

Target	Differential Expression	Peak # Location	Differential Methylation	Expected Effect of KD on Resistance	Actual Effect of KD on Resistance: CDI	Corroborates?
**FABP5**	↓	1: utr5	↑	Antagonism	0.5519 SYNERGISTIC	**OPPOSITE**
**SERPINE1**	↓	1: utr3	↑	Antagonism	0.9553	NOT SIGNIFICANT
**OSBP2**	↓	1: exon	↑	Antagonism	0.7738	NOT SIGNIFICANT
**ANO9**	↓	1: exon<br>2: exon	↑	Antagonism	0.5614 SYNERGISTIC	**OPPOSITE**
**PLA2R1**	↓	1: exon	↓	Antagonism	0.7466	NOT SIGNIFICANT
**SLC7A11**	↑	1: utr5	↓	Synergy	0.4198 SYNERGISTIC	**YES**
**PML**	↑	1: exon	↑	Synergy	0.9549	NOT SIGNIFICANT

Supplementary Table 5. Summary table of functional validation for the 7 transcripts previously validated by qPCR and MeRIP-qPCR. Arrows indicate expression or methylation levels in T24R2 compared to T24. CDI is a coefficient of synergy where CDI < 0.7 indicates statistically significant synergy and CDI > 1 indicates statically significant antagonism. CDI is the average of 3 biological replicates.

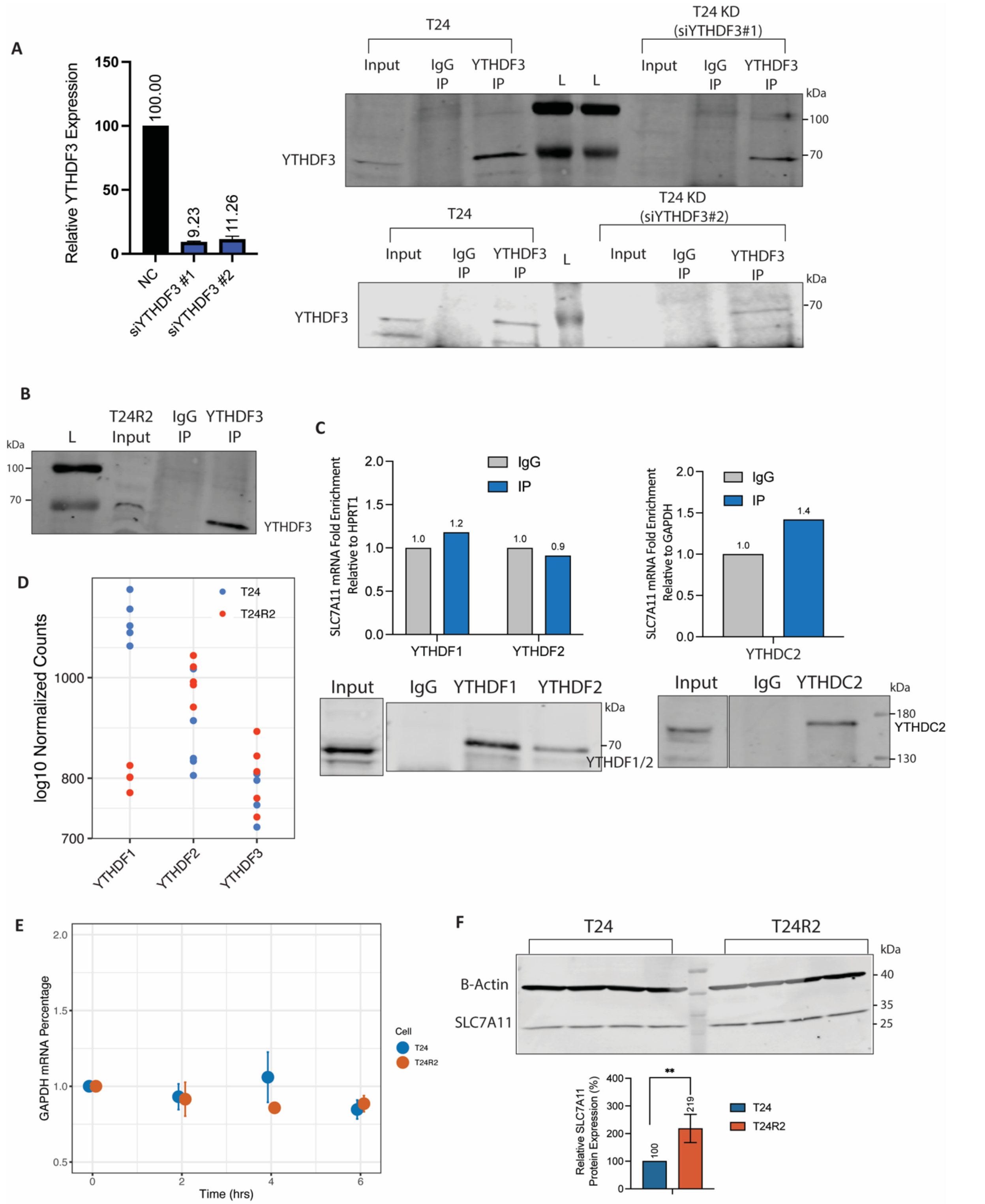

Supplementary Figure 4. Immunoprecipitation efficiency for RIP-qPCR and mRNA decay negative controls in T24 and T24R2 BC cells. Western blot showing immunoprecipitation efficiency for RIP-qPCR experiment for YTHDF3 in (A) T24 and T24 siYTHDF3 KD (two independent siRNAs) and (B) T24R2. Negative RIP-qPCR results and IP efficiency for additional m^{6}A readers, (C) YTHDF1-2 and YTHDC2 showing no enrichment of SLC7A11 mRNA upon significant pull-down. (D) Normalized RNA-seq count of YTHDF1-3 in T24 and T24R2 cells. (E) Negative control GAPDH mRNA decay in T24 and T24R2 via Actinomycin D RNA stability assay. (F) Western blot and densitometric quantification of SLC7A11 protein expression between T24 and T24R2 in four additional replicates. L=ladder.

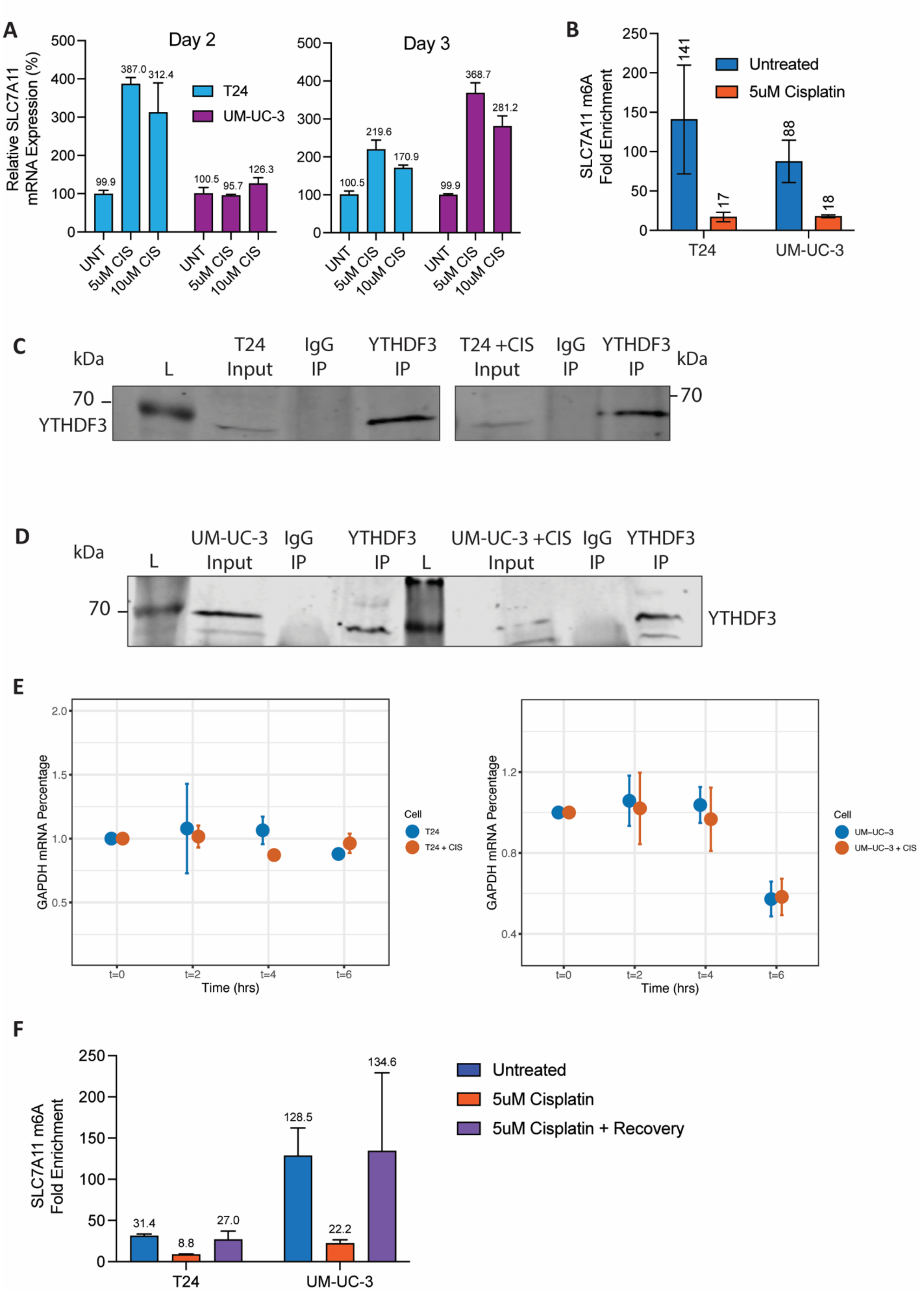

Supplementary Figure 5. Cisplatin treatment optimization, MeRIP-qPCR and immunoprecipitation efficiency for RIP-qPCR for T24 and UM-UC-3 BC cells. (A)

Relative SLC7A11 mRNA expression in T24 and UM-UC-3 cells untreated vs treated with 5uM and 10uM cisplatin after 48 and 72 hours. (B) SLC7A11 m^6A fold enrichment for T24 and UM-UC-3 untreated and treated with 5uM cisplatin for 48 hours using MeRIP-qPCR. (C-D) Western blot showing immunoprecipitation efficiency for RIP-qPCR experiment for YTHDF3 in (C) T24 +/- cisplatin and (D) UM-UC-3 +/- cisplatin including input, IgG control IP and YTHDF3 IP. (E) Negative control GAPDH mRNA decay in T24 and UM-UC-3 cells treated with cisplatin compared to untreated cells. (F) SLC7A11 m^6A fold enrichment for T24 and UM-UC-3 cells at baseline, after cisplatin treatment, and again after recovery from cisplatin treatment, using MeRIP-qPCR. L=ladder.

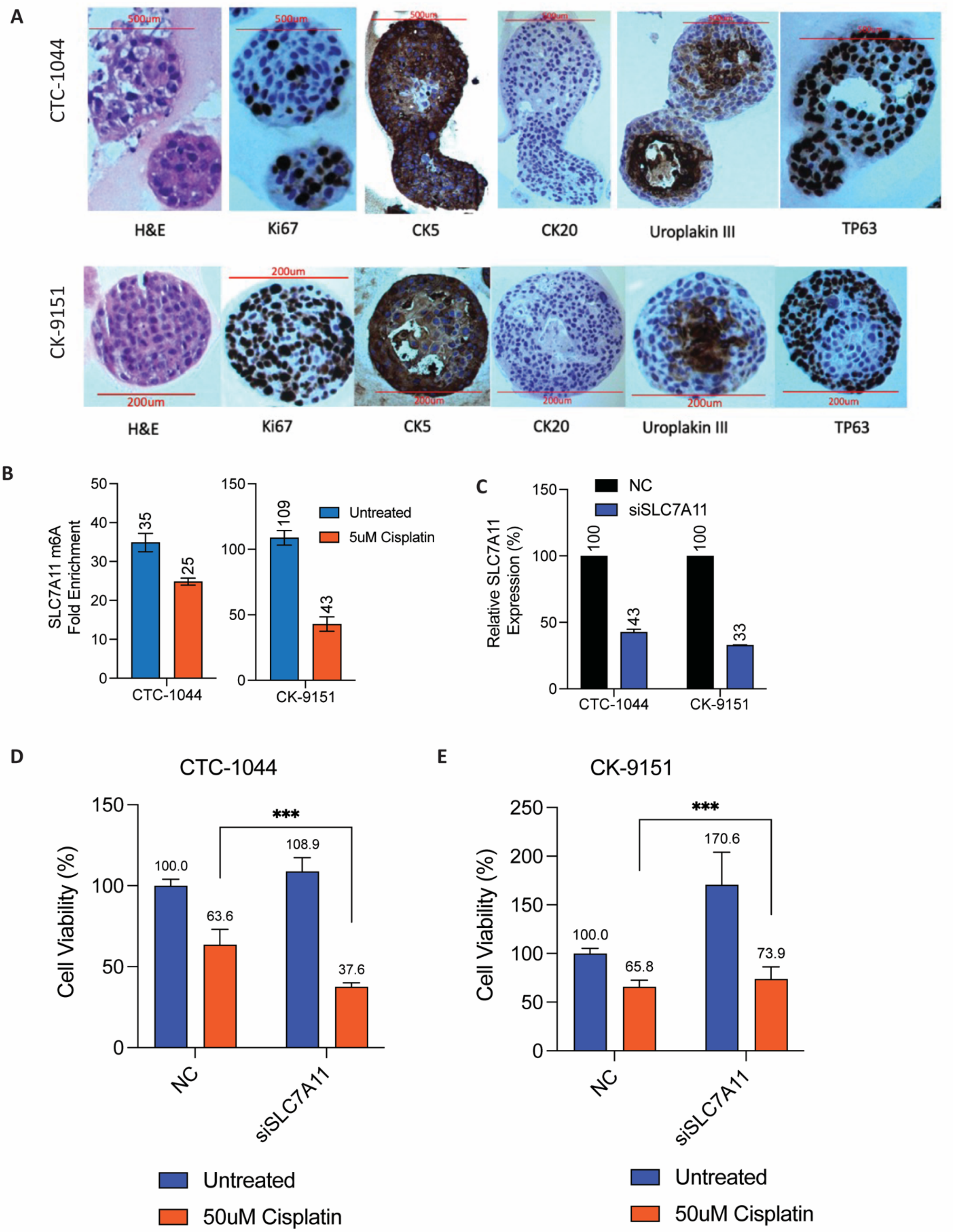

Supplementary Figure 6. Characterization, MeRIP-qPCR, SLC7A11 siRNA knockdown efficiency and cell viability of PDOs. (A) Characterization of patient derived organoids (PDOs) using histologic stains for H&E, CK5, Ki67, CK20, TP63 and Uroplakin III at 10X. (B) SLC7A11 m^6A fold enrichment by MeRIP-qPCR for PDO CTC-1044 and CK-9151 untreated vs treated with 50uM cisplatin for 48 hours. (C) SLC7A11 siRNA-knockdown efficiency in CTC-1044 and CK-9151. Cell viability upon siSLC7A11 knockdown with and without cisplatin treatment in (D) CTC-1044 and (E) CK-9151. NC= negative control. Significant codes: '***': 0.001, '*': 0.05.
